# Dynamic Chromatin States Coupling with Key Transcription Factors in Colitis‐Associated Colorectal Cancer

**DOI:** 10.1002/advs.202200536

**Published:** 2022-06-16

**Authors:** Lin Chen, Zhihui Luo, Chen Zhao, Qinglan Li, Yingjie Geng, Yong Xiao, Ming‐Kai Chen, Lianyun Li, Zhen‐Xia Chen, Min Wu

**Affiliations:** ^1^ Frontier Science Center for Immunology and Metabolism Hubei Key Laboratory of Cell Homeostasis Hubei Key Laboratory of Developmentally Originated Disease Hubei Key Laboratory of Enteropathy College of Life Sciences Renmin Hospital of Wuhan University Wuhan University Wuhan Hubei 430072 China; ^2^ Hubei Hongshan Laboratory Hubei Key Laboratory of Agricultural Bioinformatics College of Life Science and Technology College of Biomedicine and Health Interdisciplinary Sciences Institute Huazhong Agricultural University Wuhan 430070 China; ^3^ Shenzhen Institute of Nutrition and Health Huazhong Agricultural University Shenzhen 518000 China; ^4^ Shenzhen Branch Guangdong Laboratory for Lingnan Modern Agriculture Genome Analysis Laboratory of the Ministry of Agriculture Agricultural Genomics Institute at Shenzhen Chinese Academy of Agricultural Sciences Shenzhen 518000 China

**Keywords:** chromatin states, colitis‐associated cancer, histone modification, NF‐*κ*B, OTX2

## Abstract

Inflammation is one of the critical risk factors for colorectal cancer (CRC). However, the mechanisms for transition from colitis to CRC remain elusive. Recently, epigenetic changes have emerged as important regulatory factors for colitis‐associated cancer. Here, a systematic epigenomic study of histone modifications is performed, including H3K4me1, H3K4me3, H3K27ac, H3K27me3 and H3K9me3, in an AOM‐DSS‐induced CRC mouse model. In combination with transcriptomic data, the authors generate a dataset of 105 deep sequencing files and illustrate the dynamic landscape of chromatin states at five time points during inflammation‐cancer transition. Functional gene clusters are identified based on dynamic transcriptomic and epigenomic information, and key signaling pathways in the process are illustrated. This study's results reveal that enhancer state regions play important roles during inflammation‐cancer transition. It predicts novel transcription factors based on enhancer information, and experimentally proves OTX2 as a critical tumor suppressive transcription factor. Taken together, this study provides comprehensive epigenomic data and reveals novel molecular mechanisms for colitis‐associated cancer.

## Graphical abstract

The dynamic changes of chromatin states during the transition from inflammation to cancer are studied in a colitis‐associated cancer model. Multiple omics analysis reveal that enhancer state regions play important roles during inflammation‐cancer transition. Novel transcription factors, such as OTX2, are discovered as critical factors for colorectal cancer.

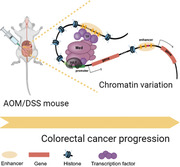

## 1 Introduction

Colorectal cancer (CRC) is one of the most common cancers in the world. CRC develops in ≈5% of the adults in the United States, and ≈50% of the patients die from the disease.^[^ ^1^ ^]^ The risk of CRC is much higher in patients with inflammatory bowel disease (IBD) than the general population, and inflammation is considered as one of the critical tumor‐promoting factors and now the mechanisms are under extensively investigation.^[^ ^2^ ^]^ Transcription factors, such as NF‐*κ*B, are highly involved in colitis‐associated CRC through promoting a localized inflammatory response and enhancing the growth and survival of tumor cells.^[^ ^3^, ^4^ ^]^ However, it remains elusive whether other transcription regulators are involved in the process.

Epigenetic features frequently change in CRC tissues and multiple epigenetic genes have been reported to be associated with CRC^[^ ^5^, ^6^ ^]^ Recently aberrant DNA methylation and regulation of enhancer activity have emerged to be important features in CRC. DNA methylomes have been extensively investigated in CRC patients and methylated DNA fragments of specific genes have been developed as diagnosis markers for CRC.^[^ ^5^, ^7^ ^]^ Genome‐wide profiling of active enhancers also turns out to be a powerful tool to identify tumor‐specific enhancers and transcription factors.^[^ ^8^, ^9^ ^]^ However, profiling of one single chromatin modification does not reflect the complete features of genome loci, and sometimes the information could be misleading. Moreover, patient samples usually hardly provide mechanistic information for disease development, and studies in animal models are urgent to reveal the underlying mechanisms.

Histone modifications are critical marks for the functions and features of certain chromatin elements. H3K4me1 is the mark for primed enhancers; H3K4me3 usually marks transcription start sites (TSS); H3K27ac is distributed on both active enhancers and promoters; H3K27me3 covers transcription repressed regions, and H3K9me3 is for heterochromatin^[^ ^10^, ^11^ ^]^ To fully understand chromatin functions, a software called ChromHMM was developed to determine chromatin states by considering the impacts of multiple chromatin modifications.^[^ ^12^, ^13^ ^]^ Although it is not perfect to describe and explain every chromatin event, it is useful to summarize the genome‐wide chromatin features and predict novel regulators. The approach has been used in the studies in cell lines, human tissues, and animal models.^[^ ^14^ ^]^ Recent studies have tried to investigate a genome‐wide enhancer state in CRC patient tissues or organoid derived from patient cells.^[^ ^15^, ^16^ ^]^ However, it is not clear how dynamic chromatin states contribute to inflammation‐cancer transition in colitis‐associated CRC.

In the current study, we performed epigenomic and transcriptome studies in an colitis‐associated CRC mouse model induced by azoxymethane (AOM) and dextran sodium sulfate (DSS).^[^ ^17^ ^]^ Combining these data, we generated the genome wide landscape of chromatin states during inflammation‐cancer transition. Our study provides important datasets for CRC studies, and reveals new regulatory mechanisms and potential targets for clinical investigation.

## 2 Results

### 2.1 Animal Model of Colitis‐Associated Cancer and Experimental Design

**Figure 1:**
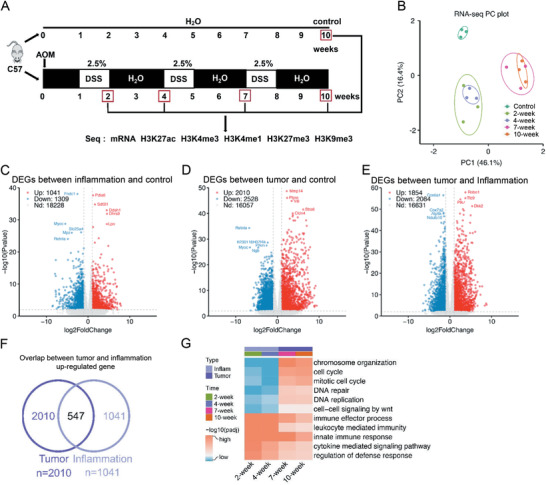
Transcriptomic and epigenomic analysis in the AOM‐DSS induced CRC model. A) Experimental workflow for studying comprehensive epigenomic landscape of colitis‐associated cancer. Each mouse was intraperitoneally injected with AOM (10 mg kg^−1^), followed by 2.5% DSS fed in feeding water. The tissues were collected and sequenced at the indicated time points. Three biological replicates were assayed for each time point. B) PCA plot of RNA‐seq data. Five time points were clustered into three stages; control, inflammation (2‐ and 4‐week), and tumor (7‐ and 10‐week). Green represents the control, light green for 2‐week, purple for 4‐week, pink for 7‐week, and orange for 10‐week. C–E) Volcano plots to show the fold change (FC) and *p*‐value of DEG expression between inflammation stage and control groups (C), tumor stage and control groups (D), and tumor and inflammation stage groups (E). A threshold of (twofold change and −log10 *p*‐value) is used for defining significant changes. Red dots represent up‐regulated genes, blue dots for down‐regulated genes, and grey dots for genes not significantly changed. F) Overlap of up‐regulated DEGs in tumor and inflammation stages. G) GSEA analysis to show the enriched pathways for DEGs of inflammation and tumor stages, in comparison with control group.

To study the epigenomic changes during inflammation‐cancer transition of colitis‐associated cancer, we used an AOM‐DSS induced colitis‐associated CRC mouse model. Colon or tumor tissues were collected at time points of 2, 4, 7, and 10 weeks after AOM injection and the control group was raised to the same time as the 10‐week group. Three mice for each time point were subjected for sequencing study (**Figure** **1**A and Figure S1A, Supporting Information). The animal body weight decreased after being fed with DSS at each round, and inflammation and tumors were observed in mouse tissues (Figure S1B–F, Supporting Information). Then RNA‐seq, and ChIP‐seq of H3K4me1, H3K4me3, H3K9me3, H3K27me3 and H3K27ac were performed with Illumina NovaSeq platform. Including input samples for ChIP‐seq, totally 105 samples were deep sequenced and the original data have been uploaded to GEO database.

### 2.2 Transcriptomic Changes during Inflammation‐Cancer Transition of Colitis‐Associated Cancer

We first determined that the sequencing depth and mapping ratios of our RNA‐seq data. The read numbers for all samples were above 20 m and their mapping rates were all ≈75%, indicating the quality was good enough for further data analysis (Figure S2A,B and Table S1, Supporting Information). We then calculated the correlation of our RNA‐seq results, which showed that samples at 2‐ and 4‐week were clustered together and those at the 7‐ and 10‐week were clustered together (Figure S2C, Supporting Information). PCA (principal components analysis) indicated the similar result (Figure 1B). Functional analysis of different expressed genes (DEGs) compared with control tissues showed that DEGs of the 2‐ and 4‐week samples were enriched in inflammation pathways, and those of the 7‐ and 10‐week samples were enriched in inflammation and cancer‐related pathways (Figure 1C–G and Figure S2D–H, Supporting Information). Based on the above, we considered that the 2‐ and 4‐week represented the inflammation stage, and the 7‐ and 10‐week for tumor stage. To confirm our deduction, we compared our data with a previous study in mouse models.^[^ ^18^ ^]^ PCA showed that our data at the 2‐ and 4‐week were grouped with their inflammatory bowel disease (IBD) samples and those at the 7‐ and 10‐week with CRC samples (Figure S2I, Supporting Information). These indicated that our transcriptomic data are reliable. Totally, our analyses identified 1041 up‐regulated and 1309 down‐regulated DEGs in inflammatory stage, and 2010 up‐regulated and 2526 down‐regulated DEGs in tumor stage (Figure 1C–E).

### 2.3 Chromatin State Dynamics during Inflammation‐Cancer Transition

To investigate the chromatin states in our model, we performed ChIP‐seq of H3K27ac, H3K4me1, H3K4me3, H3K27me3 and H3K9me3 with tissues collected at the 5 time points. The mapping rate, read number, and peak number of each sample were shown (Figure S3A–C and Table S1, Supporting Information). Correlation analysis of genome distribution showed high correlation among the same modification of 15 samples, as well as among three active transcription marks (Figure S3D, Supporting Information). PCA also grouped samples of the same modification together nicely (Figure S3E, Supporting Information). All the above indicates that our ChIP‐seq data were reliable for further analysis, and ChIP‐seq intensity of each modification indicated that all modifications changed dynamically during transition (Figure S3F–J, Supporting Information).

**Figure 2:**
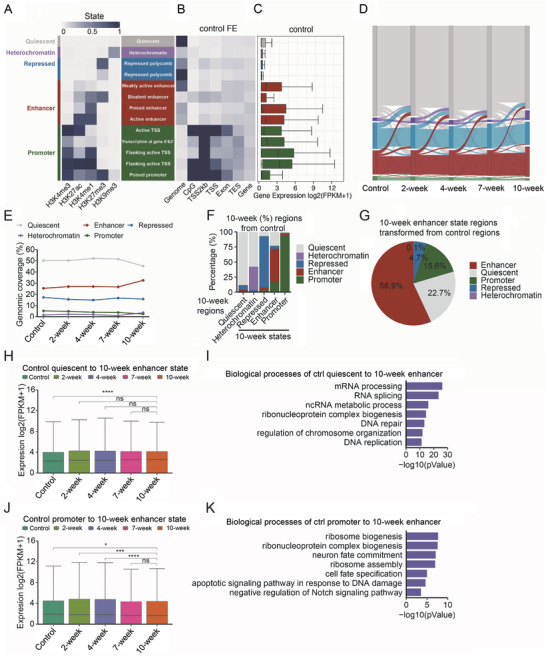
Dynamic chromatin states during inflammation‐cancer transition. A) Chromatin state definitions based on histone modification enrichment determined by ChromHMM software. The genome is divided into 13 chromatin states, which is further summarized into five chromatin states and highlighted with different colors, quiescent in grey, heterochromatin in purple, transcriptional repressed in blue, enhancer in red and promoter in green. B) Chromatin state enrichments on various genomic elements. FE means fold enrichments. C) Boxplot shows difference of all gene expression (FPKM) within particular chromatin state regions based on RNA‐seq of control tissues (gene body ± 2 kb). D) Alluvial plots showing the whole genomic dynamic changes of chromatin states during the development of Colitis‐Associated Cancer. E) Line chart shows the percentages of chromatin regions of five chromatin states at 5 time points. F) A stacked bar chart displays the percentage of each chromatin state regions at 10‐week transformed (gene body ± 2 kb) from those in control tissues. G) The percentage of 10‐week enhancer state regions originated from the chromatin state regions of control tissues. H) Dynamic expression (FPKM) of 3887 genes within the regions of control quiescent state transformed to 10‐week enhancer state. I) Biological process analysis of genes in (H). J) Dynamic expression (FPKM) of 2757 genes within the regions of promoter state regions in control transformed to 10‐week enhancer state. K) Biological process analysis of genes in (J). H,J) Wilcoxon rank sum test. (Mann–Whitney U test) ∗ *p* < 0.05, ∗∗ *p* < 0.01, ∗∗∗ *p* < 0.001, ∗∗∗∗ *p* < 0.0001, ns: no significance.

To determine the landscape of chromatin states, we defined 13 states with different combinations of modifications as below: quiescent state regions (no detected modifications), heterochromatin state regions (dominant H3K9me3), transcription repressed regions (dominant strong or weak H3K27me3), active enhancer state regions (high H3K4me1 and H3K27ac), poised enhancer state regions (high H3K4me1 and low H3K27ac), bivalent enhancer state regions (H3K4me1, H3K27ac, and H3K27me3), weakly active enhancer state regions (low H3K4me1 and H3K27ac), active promoter state regions (totally four types of regions were identified which all have H3K4me3 and are close to TSS), and poised promoter state regions (high H3K4me3 and H3K27me3) (**Figure** **2**A). The genome distribution of promoter state regions was all enriched on CpG islands and TSS, and others were widely dispersed all over the genome (Figure 2B), which fit our expectation. The expression of genes within dominant promoter state and enhancer state regions was higher than those in other regions, which also fit our expectation (Figure 2C and Figure S4A, Supporting Information). Interestingly, we found that the promoter state regions with strong H3K27ac covered CpG islands and vice versa. Since DNA methylation on CpG islands represses transcription, it probably suggests a possible link between DNA methylation and H3K27ac on CpG islands.

Then we compared the chromatin states at different time points, we found that all the above chromatin state regions were highly dynamic during inflammation‐cancer transition (Figure 2D–G and Figure S4B–L, Supporting Information). The enhancer state regions kept increasing during transition, especially at the late tumor stage (Figure 2E). Most of the quiescent, repression and promoter regions at the 10‐week were similar to control tissues, while a large percentage of enhancer state regions at the 10‐week originated from quiescent and promoter regions of control group. Moreover, many heterochromatin regions transited from quiescent state regions (Figure 2F). A pie chart showed that 22.7% of enhancer state regions at the 10‐week were originated from quiescent regions, 15.6% from promoter regions, and 4.7% from repressed regions of control tissues (Figure 2G). Genes within the 10‐week enhancer state regions transited from quiescent regions were enriched with processes related with mRNA processing and DNA repair (Figure 2H,I); and those transited from promoter regions were enriched with genes related with ribosome biogenesis, cell fate determination, and apoptotic signaling (Figure 2J,K). These suggested that acquisition of enhancer state regions is an important feature during inflammation‐cancer transition.

### 2.4 Functional Gene Sets Determined by Transcriptome‐Based Dynamic Gene Analysis

**Figure 3:**
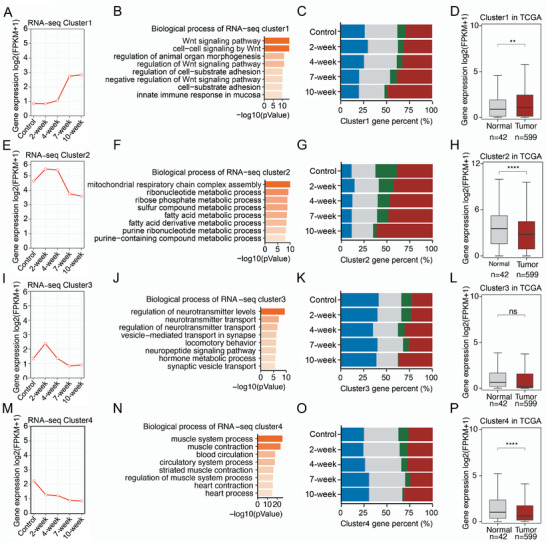
Functional gene clusters identified using dynamic transcriptomic analysis. Four cluster genes identified by dynamic transcription level at five‐time points, A–D) cluster1, E–H) cluster 2, I–L) cluster 3, and M–P) cluster 4. B,F,J,N) Biological process analysis of the 4 gene clusters. Bar plot gradient color fill with the *p*‐value. C,G,K,O) The proportion of 5 chromatin states of the genes in four clusters at different time points. Quiescent: grey, heterochromatin: purple, repressed: blue, enhancer: red, promoter: green. D,H,L,P) Boxplots show expression (FPKM) of the 4 cluster genes in human CRC (*n* = 599) and normal (*n* = 42) tissues. The RNA‐seq datasets of human colorectal cancer (COAD) were downloaded from TCGA database. Statistical analysis was performed using an unpaired student's *t* test. ∗ *p* < 0.05, ∗∗ *p* < 0.01, ∗∗∗ *p* < 0.001, ∗∗∗∗ *p* < 0.0001, ns: no significance.

Although we analyzed the functions of DEGs, it was still difficult to obtain a comprehensive picture of the dynamic change during inflammation‐cancer transition. So, we used maSigPro software to determine functional gene clusters based on their dynamic expression levels. We successfully divided all dynamic genes into four clusters (**Figure** **3**A–P and Table S2, Supporting Information), and the intensity of histone modifications of each cluster changed dynamically (Figure S5A–D, Supporting Information). Genes of cluster 1 expressed low in normal and inflammatory stages and increased dramatically in cancer stage (Figure 3A). They were enriched in cancer‐related pathways, especially WNT signaling pathway and immune response (Figure 3B). Enhancer state regions were significantly enriched on these genes, and all the other states decreased (Figure 3C). According to TCGA data, the orthologues of cluster 1 genes expressed higher in tumor tissues than normal tissues in patients (Figure 3D). Genes of cluster 2 increased their expression at the 2‐week, maintained at the inflammation stage and decreased at the tumor stage (Figure 3E). These genes were enriched in metabolic pathways (Figure 3F). Genes of cluster 3 increased at the 2‐week and then gradually decreased later (Figure 3I), and were enriched in the processes related with transportation and inflammation (Figure 3J). Enhancer state regions also increased for genes of cluster 2 and 3 (Figure 3G,K); and the expression of their human orthologues decreased in tumor tissues according to TCGA data (Figure 3H,L). Genes of cluster 4 decreased during all the stages and were enriched in processes involved in normal tissue functions, such as muscle contraction and blood circulation (Figure 3M,N). The expression of their orthologue genes also decreased in tumor tissues (Figure 3P). The transcription repression state regions on them increased but enhancer regions did not, which was different from the above three clusters (Figure 3O).

### 2.5 Functional Gene Clusters Identified with H3K27ac on Enhancers

**Figure 4:**
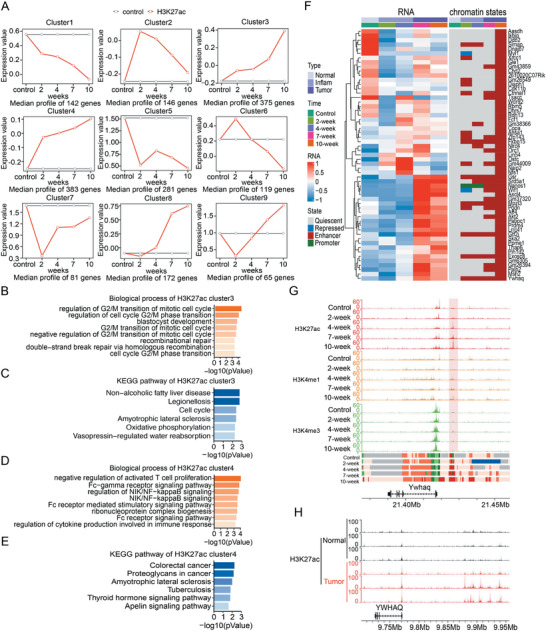
Identification of functional gene clusters based on dynamic H3K27ac signal. A) We calculated H3K27ac enrichment (RPM) on enhancer region for each gene on TSS ± 10 kb to TSS ± 1.5 kb. Then we used maSigPro software and divided these genes into 9 clusters by dynamic H3K27ac signal. The control group data were as control, others as treatment. Each group had 3 biological replications. Red lines represented H3K27ac signal at 4 time points (2, 4, 7, and 10‐week). Grey lines represented control group data. The enriched genes were identified by R after scaling count data. Expression value was evaluated by maSigPro. B–E) Biological process and KEGG analyses of genes in cluster 3 and 4, by R package clusterProfiler. F) Gene expression heatmap (left) and its corresponding chromatin state (right) of cluster 3 genes whose chromatin states were transit from quiescent in control to enhancer at 10‐week. G) The UCSC browser view shows H3K27ac, H3K4me1, and H3K4me3 enrichments around *Ywhaq*. H) The UCSC browser view for H3K27ac enrichment around *YWHAQ* of 3 pairs of normal and colon tumor patient tissues.

We successfully clustered dynamic genes into four clusters with distinguished functions, then we explored whether we could classify the genes into functional gene sets with dynamic chromatin states or histone modifications. Since it is difficult to quantify chromatin states, we applied the above analysis based on the RPM (reads per million) values of histone modifications on genes. Because histone modifications around gene body may also regulate gene expression, it is better to integrate the signals on gene bodies and regions close to them. After comparing the analytical results of different ranges, we used the signal within gene body flanked ±2 kb for the following analyses; while for analysis of histone modifications on enhancers, signals close to TSS were excluded (see the Experimental Section for details). All the above modifications were investigated and we found that the classification with H3K27ac on enhancers was mostly meaningful. It supports the recently raised hypothesis that enhancer activation is one of the important features for cancer^[10,^ ^19^ ^]^ The dynamic genes were divided into 9 clusters according to their H3K27ac signal on enhancers (**Figure** **4**A and Table S3, Supporting Information). Since the above analysis suggested the increase of enhancer state regions was associated with inflammation‐cancer transition, we focused on the functions of three clusters 3, 4 and 8, which showed elevated H3K27ac during transition (Figure 4A–E and Figure S6A–N, Supporting Information). Genes of cluster 3 were enriched in cell cycle and DNA repair processes, closely related with cancer (Figure 4B,C). Genes of cluster 4 were enriched in inflammation and cancer related processes, such as NF‐*κ*B pathway (Figure 4D,E), which has been proved to be critical for colitis‐associated CRC.^[^ ^4^ ^]^ Genes of cluster 8 were enriched in processes related with lipid metabolism and PKA signaling, which is also involved in CRC (Figure S6F,M, Supporting Information).^[^ ^20^ ^]^ Our analysis suggested that the information of histone modifications on function chromatin elements can be used to identify functional gene sets. The biological processes and pathways enriched in other clusters may be also informative to CRC studies.

We used the genes of cluster 3 to further investigate the relationship between gene expression and chromatin states. The dominant chromatin states on a gene (gene body ± 2 kb) were used to represent its chromatin states. A large portion of genes of cluster 3 showed a high level of enhancer state, and we took those showing conversion from quiescent to enhancer state to investigate the relationship between chromatin states and gene expression. Although the expression of some genes was not always correlated with their dominant chromatin state, most of them showed high expression at the cancer stage (7‐ or 10‐week), suggesting the elevated H3K27ac on these genes were associated with tumorigenesis (Figure 4F). These suggested chromatin state analysis could provide additional information other than transcriptomic studies. Tyrosine 3‐monooxygenase/tryptophan 5‐monooxygenase activation protein (*Ywhaq*, 14‐3‐3) is a cancer‐related gene^[^ ^21^ ^]^ and selected as a representative to show the dynamic change of chromatin states and histone modifications (Figure 4G). H3K27ac signal on the enhancer of *YWHAQ* in CRC patient samples also increases compared with the adjacent tissues (Figure 4H).^[^ ^16^ ^]^

### 2.6 Chromatin State Dynamics of WNT Signaling Target Genes

WNT signaling is an important pathway in CRC. Our transcriptomic analysis using maSigPro revealed cluster 1 genes were enriched in the WNT signaling pathway (Figure 3B). To investigate the potential functions of dynamic chromatin states in CRC, we took the genes of cluster 1 belonging to the WNT pathway (totally 44 genes) and analyzed their relationship with chromatin states. Out of the 44 genes, 12 genes were enriched with a dominant enhancer state in control tissues, then the number increased to 24 genes at 10‐weeks. For other states, such increase was not observed (Figure S7A, Supporting Information). Analysis of histone modification intensity showed that H3K27ac signal increased during inflammation‐cancer transition on these genes (Figure S7B, Supporting Information). The 24 genes with dominant enhancer state at the 10‐week had higher expression in CRC tumor tissues according to TCGA data (Figure S7C, Supporting Information). Among the above 24 genes, 11 genes kept their dominate enhancer state from control to 10‐weeks, and others were transited from other chromatin states (Figure S7D, Supporting Information). *AXIN2* is a typical WNT pathway target gene and was used as a representative gene to show its expression, histone modifications, and chromatin states during transition (Figure S7E–G, Supporting Information). Our recent study in CRC patient samples also showed its H3K27ac around *AXIN2* (Figure S7H, Supporting Information).^[^ ^9^ ^]^ These suggest that enhancer chromatin states are involved in the regulation of WNT target genes in colitis‐associated CRC.

### 2.7 Enhancer State Regions Are Correlated with Expression of NF‐*κ*B Target Genes

**Figure 5:**
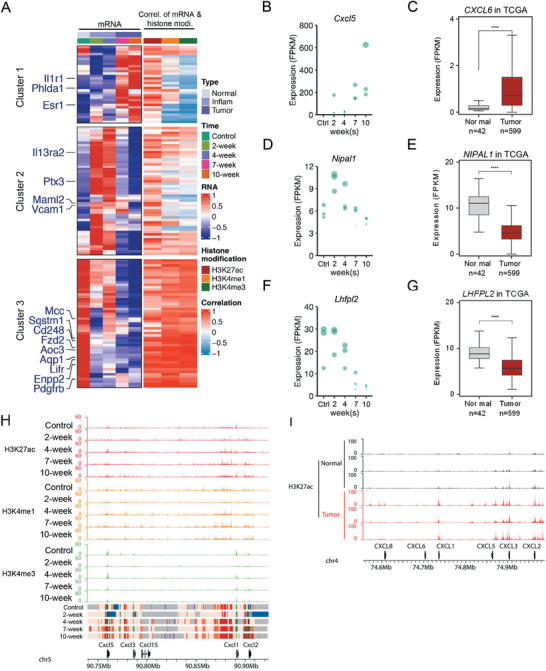
Enhancer state regions are associated with selectively activation of NF‐*κ*B target genes. A) The heatmap (left) shows the gene expression regulated by NF‐*κ*B signal pathway at five time points. Correlations between dynamic gene expression and the corresponding H3K27ac, H3K4me1, and H3K4me3 signals (RPM, gene body ± 10–100 kb) (right panel) were calculated, respectively. Genes reported to be involved in CRC were marked in blue. B–G) Gene expressions of representative genes for each cluster (B,D,F), and the corresponding gene expression in TCGA database (C,E,G). H) The UCSC browser view shows H3K27ac, H3K4me1, and H3K4me3 enrichment on *Cxcl* family locus. I) The UCSC browser view for H3K27ac enrichment of 3 pairs of normal and colon tumor patient tissues around *CXCL* family locus. An unpaired student's *t* test was used. ∗ *p* < 0.05, ∗∗ *p* < 0.01, ∗∗∗ *p* < 0.001, ∗∗∗∗ *p* < 0.0001, ns: no significance.

The NF‐*κ*B transcription factor plays critical roles in colitis‐associated CRC.^[^ ^4^ ^]^ In our analysis of gene clusters with dynamic H3K27ac, we found that genes of cluster 4 were enriched in NF‐*κ*B signaling pathway (Figure 4D). To further investigate the relationship between NF‐*κ*B and chromatin states during inflammation‐cancer transition, we analyzed the dynamics of NF‐*κ*B downstream genes identified from one of our previous studies (**Figure** **5**A and Figure S8A, Supporting Information).^[^ ^22^ ^]^ Interestingly, gene clustering analysis clearly divided the overlapped genes into three groups, which were specifically expressed in three stages (Figure 5A, left panel, and Figure S8B–D, Supporting Information). It indicated that NF‐*κ*B regulated distinct transcription programs in the three stages, and transition of NF‐*κ*B target genes might be important for inflammation‐cancer transition. To investigate whether chromatin states or histone modifications were involved, we calculated the correlation of H3K27ac, H3K4me1, and H3K4me3 with the dynamic gene expression level of each gene, respectively. For cluster 1 and 2, whose expression were highest at tumor or inflammation stage, H3K27ac had the highest correlation; for cluster 3, whose expression was highest in normal tissues, all three modifications showed relative high correlation (Figure 5A, right panel). C‐X‐C motif chemokine ligand 5 (Cxcl5), NIPA‐like domain containing 1 (Nipal1), and lipoma HMGIC fusion partner‐like 2 (Lhfpl2) were selected as the representative genes of each cluster to show their expression at 5 time points and their orthologues in patient samples (Figure 5B,C). Chromatin state analysis showed that among all the dynamic genes regulated by NF‐*κ*B, only those within enhancer state regions showed an increasing trend during transition, which further supported the important roles of enhancer state (Figure S8E, Supporting Information). Among them, 39.6% genes were transited from quiescent state genes (Figure S8F, Supporting Information). These results suggest that H3K27ac dynamics on enhancers is associated with transition of NF‐*κ*B target genes.

### 2.8 Predictions of Functional Transcription Factors with Enhancer Regions

**Figure 6:**
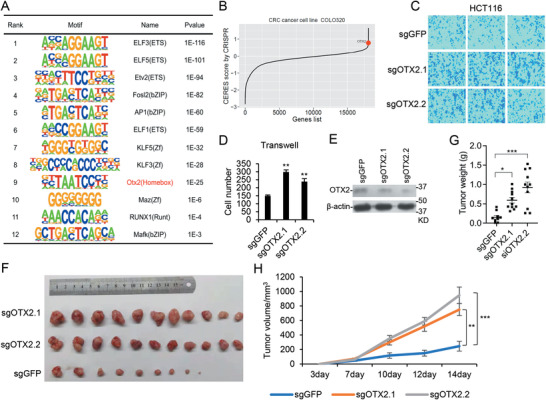
Identification of Otx2 as a tumor suppressive transcription factor. A) The top 10 TFs enriched on variant enhancers of 10‐week versus control, and TFs selected for experimental validation. B) CERES score of OTX2 in COLO320 cell line. C–E) *OTX2* was knocked down by sgRNA in HCT116 cells. The typical images (C) and statistical analysis (D) of cell migration, and OTX2 protein level (E) were shown. The results represent the means (± SD) of at least three independent biological replicates. F–H) *OTX2* stably knockdown HCT116 cells used in above experiments were injected into nude mice (1 × 10^6^ cell pear mouse, *n* = 11). Tumors were pictured (F), and tumor weight (G) and growth curve (H) were shown as mean (± SEM). Statistical analysis was performed using ordinary one‐way ANOVA (G) plotted by software prism9 and an unpaired student's *t* test (H) by R. ∗ *p*‐value ≤ 0.05, ∗∗ *p*‐value ≤ 0.01, ∗∗∗ *p*‐value ≤ 0.001.

Transcription factors (TFs) play key roles in multiple biological processes, and their functions are tightly related with enhancer activation and regulation. To further explore the molecular mechanisms of colitis‐associated cancer, key TFs specifically involved in each stage were analyzed by motif analysis with the corresponding enhancer regions. The top 10 TFs enriched on variant enhancers of the 10‐week versus control, together with the TFs validated experimentally, are shown (**Figure** **6**A, and Figure S9A–C and Table S4, Supporting Information). The top TFs were enriched in several protein families, such as AP‐1 and ETS families, whose functions in cancer have been well established. We selected ≈10 TFs for validation, which were not well characterized, and tried to knock them down with siRNAs. We successfully knocked down *OTX2*, *RUNX1*, *MAZ*,s and *MAFK* in HCT116 colorectal cancer cells, respectively. For other TFs, either their expression levels were very low in cells, or siRNAs did not work (Figure S9, Supporting Information). We then explored their functions in CRC experimentally. Knockdown of *MAZ* or *RUNX1* impaired cell migration, indicate they both contained oncogenic functions, which are consistent with previous publications;^[^ ^23^ ^]^ and *MAFK* knockdown had no effects on either proliferation or migration.

CERES score has been used to predict the potential roles of a gene in cancer,^[^ ^24^ ^]^ and we found that *OTX2* might play a tumor suppressive role (Figure 6B). From the predicted TF table, we also found that the score of Otx2 was high in tumor stages (Table S4, Supporting Information). We further found that knockdown of *OTX2* in HCT116 with sgRNAs enhanced cell migration ability but did not affect proliferation significantly (Figure 6C–E). To confirm it, we knocked down *OTX2* in RKO cells with CRISPR, or in HCT116 cells with siRNAs. The same phenotypes were observed (Figure S10A–I, Supporting Information). Then xenograft experiments were performed with HCT116 cells containing *OTX2* sgRNAs. The results indicated that *OTX2* deficiency enhanced tumor formation of HCT116 (Figure 6F–H). These data together demonstrated that OTX2 is a tumor suppressive TF in CRC.

### 2.9 Otx2 Represses Tumorigenesis through IFITM Family Genes

**Figure 7:**
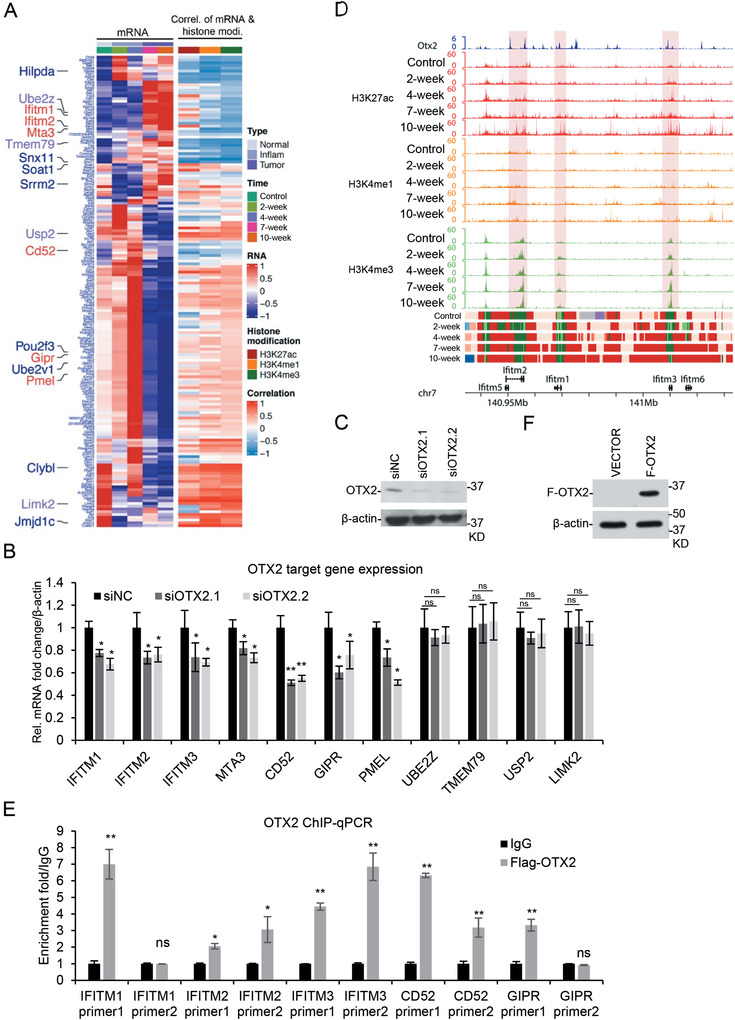
OTX2 regulates *IFITM* family genes in colitis‐associated CRC. A) Heatmap (left) to show the expression of potential Otx2 target genes at five time points. Pearson correlation coefficient between dynamic gene expression and the corresponding H3K27ac, H3K4me1, and H3K4me3 signals (right) were calculated, respectively. Genes reported to be involved in CRC were marked in blue, and genes confirmed as Otx2 target genes were marked in red. B) Expression of potential OTX2 target genes was measured in *OTX2* knockdown HCT116 cells. The results represent the means (± SD) of at least three independent biological replicates. Statistical analysis was performed using an unpaired Student's *t* test. ∗ *p* < 0.05, ∗∗ *p* < 0.01, ns: no significance. C) OTX2 protein level assayed with western blotting after knockdown by siRNAs. D) The UCSC browser view to show Otx2 binding, H3K27ac, H3K4me1, and H3K4me3 enrichment around *Ifitm* family locus. E) Flag‐OTX2 was stably expressed in HCT116, and ChIP analysis was performed with anti‐Flag antibody and assayed with quantitative PCR. F) Expression of Flag‐OTX2 assayed with western blotting.

To investigate the potential mechanisms for OTX2 in CRC, we used OTX2 ChIP‐seq data from a previous report.^[^ ^25^ ^]^ Since no Otx2 ChIP‐seq experiment were conducted on mouse colon or human colon tissue, we selected the ChIP‐seq data generated from mouse embryonic stem cells by Buecker et al. OTX2 target genes for this data had been detected by Cistrome Data Browser. We downloaded and used the top 200 genes as potential Otx2 target genes in mouse CRC, among which 171 genes were finally used in the assay after being merged with our result (**Figure** **7**A). Interestingly, the expression of Otx2 target genes also showed periodic expression at three stages, suggesting it might be a common phenotype for functional transcription factors involved in colitis‐associated cancer. We then verified the expression of the predicted target genes in human CRC cells. We found that the mRNA of *IFITM1*, *IFITM2*, *IFITM3*, *MTA3*, *PMEL*, *GIPR*, and *CD52* decreased after *OTX2* knockdown (Figure 7B,C). Our prediction of Otx2 binding sites suggested it was recruited to the above genes (Figure 7D). To investigate whether OTX2 directly regulates the above genes, we established a Flag‐OTX2 stable cell line from HCT116, and performed ChIP‐PCR study (Figure 7E,F). The result indicated that Flag‐OTX2 was bound to *IFITM1*, *IFITM2*, *IFITM3*, *GIPR*, and *CD52. IFITM* family genes are localized together in a genome locus (Figure 7D). They are involved in the suppression of immune response, and their deficiency leads to increased susceptibility to colitis; but controversial evidence exists for their roles in colorectal cancer.^[^ ^26^, ^27^ ^]^ Our data suggest that Otx2 possibly represses colitis‐associated CRC through mediating the transcription of tumor suppressive genes, such as *IFITM* family genes.

## 3 Discussion

Inflammation is one of the critical risk factors for cancer, but the mechanisms of how the inflammatory state promotes tumorigenesis in colitis‐associated CRC remain elusive. In the current study, we perform transcriptomic and epigenomic studies and reveal the dynamic landscapes of chromatin states during inflammation‐cancer transition in a colitis‐associated CRC mouse model. We discover that dynamic chromatin states are critical and enhancer state regions increase significantly during transition, which are associated with gene transcription regulated by key signaling pathways, such as WNT, NF‐*κ*B and metabolic pathways. These indicate that enhancer activation plays important roles in CRC, which supports the hypothesis raised recently that acquisition of active enhancers and H3K27ac elevation are important features for cancer.^[^ ^10^, ^19^, ^28^ ^]^

Our transcriptomic analysis using maSigPro for different stages divides the dynamic genes into four groups with distinct functions, including cancer‐related genes, metabolic genes, inflammatory genes and intestine functional genes. Cancer‐related genes are low at inflammation stage and high at cancer stage, as expected. Intestine functional genes keep decreasing at both stages, indicating the transformation of intestine normal cells to tumor cells. Metabolic genes, transportation and inflammation genes increase at inflammation stage but decrease at cancer stage, suggesting besides inflammation, change of metabolic program and regulation of material transportation are also important for colitis‐associated CRC. These provide a comprehensive picture for functional genes involved in colitis‐associated CRC.

We also identify important gene clusters using maSigPro based on dynamic H3K27ac on genes, which divided genes into 9 clusters. Genes in cluster 3, 4, and 8 showed increasing trends at inflammation and cancer stages. The three clusters were enriched with genes regulating cell cycle, NF‐*κ*B signaling, and lipid metabolism. Genes of cell cycle regulation are critical for tumor cell proliferation, and NF‐*κ*B is known to be a key TF for colitis‐associated CRC.^[^ ^4^ ^]^ Lipid metabolism has been shown recently to be critical for tumorigenesis. Our study showed the feasibility to use epigenomic data to identify functional pathways in biological processes. Beyond the above, our analysis identified multiple genes and pathways potentially involved in the process, such as JAK‐STAT pathway, PKA pathway and metabolic pathways of amino acids (Figure S6, Supporting Information). These provide important candidate genes and pathways for future studies, and indicated that epigenomic information can also be used to identify functional gene clusters. Though enhancer activation is tightly associated with transcription activation, its activation does not always lead to gene expression. Enhancer profiling provides one more layer of information for identifying functional pathways and genes.

Besides H3K27ac, we also tried to perform the similar analysis with other modifications. We just show the analyzed results of enhancer state, because we feel enhancer state dynamics show higher correlation with gene expression than others. The reason might be that H3K27ac on enhancers is more sensitive to signaling variation, or the ChIP‐seq data for H3K27ac is relatively better than others. If one method could be developed to quantify chromatin states, then it will be very powerful to annotate chromatin functions by combining the two approaches together.

Combining transcriptomic and epigenomic data, we predicted the potential target genes of NF‐*κ*B in our mouse model. Interestingly, NF‐*κ*B target genes show a periodic expression pattern and are selectively activated at inflammation and cancer stages. It needs to be determined whether the pattern is due to the different composition of cell types at different stages, or different transcriptional programs in the same cells. Nevertheless, it is important to investigate the exact roles and the underlying mechanisms for NF‐*κ*B at different stages, whether other factors function through the similar ways, and the functions of epigenetic factors in regulating the process.

Using the information about the dynamic enhancer regions at different stages, we further predicted multiple TFs, and then identified Otx2 as one novel TF involved in CRC, which plays a tumor‐suppressive function. *IFITM* family genes are localized closely in one genome locus. They act as a suppressor of the immune response and are involved in CRC. In most of the cell‐based studies, *IFITM* genes behave as oncogenes, and their deficiency impaired cell growth and invasion^[^ ^26^, ^29^ ^]^ However, in an *IFITM3* knockout model induced by AOM/DSS, its deletion led to enhanced colitis and enlarged tumor number and size, suggesting it is a tumor suppressor.^[^ ^27^ ^]^ Then it is possible that *IFITM* family genes play opposite roles in different types of CRC. In our study, we used AOM/DSS to induce colitis‐associated CRC model, so our study implies that Otx2 represses colitis‐associated CRC through regulating gene expression, such as *IFITM* family genes. Then our study provides novel mechanistic insights for the research of colitis‐associated CRC.

Taken together, our work provides an important resource for studying epigenetic regulation in colitis‐associated colorectal cancer, revealing the potential functions of enhancer states regions in NF‐*κ*B‐dependent selective transcription during inflammation‐cancer transition, and identifies the tumor suppressive functions of OTX2 in CRC. All these provide important information for future studies in colitis‐associated colorectal cancer.

## 4 Experimental Section

### Reagents and Cell Lines

Antibodies recognizing H3K4me3 (Millipore, 04–745, RRID: AB_1 163 444), H3K4me1 (CST, 5326, RRID: AB_10 695 148), H3K27me3 (clone C36B11, CST 9733, RRID: AB_2 616 029), H3K9me3 (Abcam, ab176916, RRID: AB_2 797 591), H3K27ac (Abcam, ab4729, RRID: AB_2 118 291), and OTX2 (Abclonal, A4351, RRID: AB_2 863 244) were purchased from indicated commercial sources. Protein G‐Sepharose beads (GE Healthcare), AOM (Sigma‐Aldrich, 25843‐45‐2), and DSS (MP Biomedicals, 160 110) were purchased from the indicated companies. PCR primers were custom synthesized by BGI and siRNAs by GenePharma. HCT116 and RKO Cell lines were purchased from Cell Bank of Chinese Academy and cultured under recommended conditions according to the manufacturer's instruction with 10% FBS.

### Animal Housing and Ethics Approval

Mice were purchased from Beijing HFK Bioscience. All the mice were born and maintained under pathogen‐free condition at ≈20–24 ℃ with a humidity of ≈40–70% and a 12/12‐hours dark/light cycle (lights on at 7:00 AM, lights off at 7:00 PM), with free access of water and food (Animal Center of College of Life Sciences, Wuhan University).

All the animal operations were following the laboratory animal guidelines of Wuhan University and were approved by the Animal Experimentations Ethics Committee of Wuhan University (Protocol NO. 14110B). No patient study was involved and the consent to participate is not applicable.

### Generation of Colitis‐Associated Colorectal Cancer Mice Model

The 8‐week‐old C57BL/6J male mice were randomly divided into four experimental groups (2 weeks, 4 weeks, 7 weeks, 10 weeks) and one control group (*n* = 5 per group). Mice in the experimental groups were given a single intraperitoneal injection of AOM (10 mg kg^−1^ body weight). Seven days after the AOM injection, the mice were given 2.5% DSS (w/v) in drinking water for 7 days. Then mice of the 2‐week group were sacrificed. The other three groups were fed with distilled water for 14 days and then the 4‐week group was sacrificed. The DSS/water cycle was repeated and mice were sacrificed at the 7‐ and 10‐week, respectively. The control group mice were fed with distilled water for 70 days and sacrificed. The colorectal tissues were divided to distal, middle and proximal fragments, and the distal colon fragments were collected for experiments. Tumors were observed in the mice of the 7‐ and 10‐week groups and only tumors were collected for further analysis for the two groups. A small number of mice did not survive randomly after the whole procedure, then we choose 3 mice of each group for further analysis.

### ChIP Assay

ChIP assay was performed as previously described.^[^ ^30^ ^]^ Briefly, ≈60 mg of each tissue were cut into 1 mm^3^ pieces in PBS with protease inhibitor. Tissue pieces were cross‐linked for 10 min at room temperature in 1% formaldehyde and then quenched with 0.125 m of glycine for 5 min. Cross‐linked tissues were triturated for 30s and then centrifuged at 12 000 rpm, 4 °C for 5 min. Supernatant with massive oil was discarded and the precipitates were lysed with 1 mL lysis buffer (50 mm Tris‐HCl pH 8.0, 0.1% SDS, 5 mm EDTA) for 4 min with gentle rotation. After centrifugation at 12 000 rpm, 4 °C for 2 min, the pellet was washed once with digestion buffer (50 mm Tris‐HCl pH 8.0, 1 mm CaCl2, 0.2% Triton X‐100), incubated in 630 µL digestion buffer with 1 µL MNase (NEB, M0247S) at 37 °C for 20 min, and quenched with 8 µL 0.5 m EDTA. The resulted mixture was sonicated and the pellet was discarded after centrifugation. 30 µL supernatant was taken for checking the efficiency of digestion. Immunoprecipitation was performed with 150 µL sheared chromatin, 2 µg antibody, 50 µL Protein G beads, and 800 µL dilution buffer (20 mm Tris‐HCl pH 8.0, 150 mm NaCl, 2 mm EDTA, 1% Triton X‐100, 0.1% SDS) overnight at 4 °C. Next day, the beads were washed once with Wash buffer I (20 mm Tris‐HCl pH 8.0, 150 mm NaCl, 2 mm EDTA, 1% Triton X‐100, 0.1% SDS), once with Wash buffer II (20 mm Tris‐HCl pH 8.0, 500 mm NaCl, 2 mm EDTA, 1% Triton X‐100, 0.1% SDS), once with Wash buffer III (10 mm Tris‐HCl pH 8.0, 250 mm LiCl, 1 mm EDTA, 1% Na‐deoxycholate, 1% NP‐40), and twice with TE (10 mm Tris‐HCl pH 8.0, 1 mm EDTA). The beads were eluted twice with 100 µL elution buffer (1% SDS, 0.1 m NaHCO3, 20 mg mL^−1^ Proteinase K) at room temperature. The elution was incubated at 65 °C for 6 h and then purified with the DNA purification kit (TIANGEN DP214‐03). Primers for ChIP‐qPCR are listed in Table S5, Supporting Information.

### Library Preparation for ChIP‐Sequencing

ChIP‐seq libraries were constructed with ChIP and input DNA using VATHS Universal DNA Library Prep Kit for Illumina (Vazyme ND606). Briefly, 50 µL of DNA (8–10 ng) was end‐repaired for dA tailing, followed by adaptor ligation. Each adaptor was marked with a barcode of 8 bp DNA. Adaptor‐ligated DNA was purified by AMPure XP beads (1:1) and then amplified by PCR of 9 cycles with the primer matching with adaptor universal part. Amplified DNA was purified again using AMPure XP beads (1:1) in 35 µL EB elution buffer. For multiplexing, libraries with different barcodes were mixed with equal molar quantities (30–50 million reads per library). Libraries were sequenced by Illumina Nova‐seq platform with pair‐end reads of 150 bp.

### RNA‐Sequencing

RNA extraction was performed using Ultrapure RNA Kit (CWBIO, CW0581M). Briefly, ≈40 mg tissues were triturated for 30 s in 1 mL TRIzon provided in the kit, incubated at room temperature for 5 min, added with 200 µL chloroform and shaken drastically. After centrifugation at 12 000 rpm, 4 °C for 10 min, the upper water phase was moved into an adsorption column provided by the kit. The column was then eluted with 50 µL RNase‐free water. RNA‐seq libraries were constructed by NEBNext Poly(A) mRNA Magnetic Isolation Module (NEB E7490) and NEBNext Ultra II Non‐Directional RNA Second Strand Synthesis Module (NEB E6111). mRNA was purified with poly‐T magnetic beads and first and second strand cDNA was synthesized. The resulting cDNA was purified by AMPure XP beads (1:1) and eluted in 50 µL nucleotide‐free water. The subsequent procedures were the same as described in ChIP‐seq library construction, except that the sequencing depth was 20 million reads per library. RNA‐seq libraries were sequenced by Illumina Nova‐seq platform with pair‐end reads of 150 bp.

### ChIP‐Seq Data Analysis

ChIP‐seq raw fastq data used FastQC (version 0.11.5, https://www.bioinformatics.babraham.ac.uk/projects/fastqc/) to quality control. Clean data were obtained by removing adapter with Cutadapt (version 1.16, http://cutadapt.readthedocs.io/en/stable/guide.html, parameters were “‐u 4 ‐u ‐35 ‐U 4 ‐U ‐35 ‐m 30”). Cleaned reads were aligned in paired‐end mode to mouse UCSC reference genome mm10 with BWA mem^[^ ^31^ ^]^ (version 0.7.15, http://bio‐bwa.sourceforge.net). Duplicate reads were removed by samtools rmdup (version 1.4.1, https://github.com/samtools/).^[^ ^32^ ^]^ Only unique mapped reads were used in following work. Peaks were called by MACS2 (version 2.1.1, https://github.com/taoliu/MACS, with parameters “–nomodel –keep‐dup all ‐p 1E‐10 –broad –broad‐cutoff 1E‐10 –extsize 147”) at false discovery rate (FDR) < 0.01.^[^ ^33^ ^]^

### RNA‐Seq Data Analysis

RNA‐seq cleaned fastq data were obtained in the same way as ChIP‐seq data (parameters were “‐u 4 ‐u ‐10 ‐U 4 ‐U ‐10 ‐m 30”). Quality control is done with FastQC (version 0.11.5, https://www.bioinformatics.babraham.ac.uk/projects/fastqc/) and Multiqc (version 1.7, https://multiqc.info/docs/#examples). Cleaned reads were aligned against the mouse UCSC mm10 genome with TopHat2 (version 2.1.1, http://ccb.jhu.edu/software/tophat/index.shtml).^[^ ^34^ ^]^ The gene annotation file was used UCSC mm10 genome. Differential expression genes were calculated by Bioconductor package DESeq2 (version 1.30.1, https://bioconductor.org/packages/release/bioc/html/DESeq2.html) at a fold change > 2 and *p*‐value less than 0.01.^[^ ^35^ ^]^ The gene expression level was normalized as fragments per kilobase of bin per million mapped reads (FPKM) by Cufflinks (version 2.2.1, http://cole‐trapnell‐lab.github.io/cufflinks).^[^ ^36^ ^]^

### Chromatin State Annotation

To capture histone modifications combination, we used ChromHMM (version 1.11) to get chromatin state by training a hidden Markov model.^[^ ^12^ ^]^ The mapped bed files of all five histone modification markers (H3K27ac, H3K4me1, H3K4me3, H3K27me3, and H3K9me3) across five CRC development stages was binned into non‐overlapped 200‐bp intervals using Bedtools software (version 2.29.2). The whole‐cell extract signal (input alignment files) were used as control data, providing background signal level to adjust the binarization criteria. The model was trained with a range of chromatin states from 10–15, and finally selected the 13 states model in subsequently analysis as it could sufficiently capture the key interactions between all histone modification markers (Figure 2A). With the chromatin state pattern and the enrichment of each state on genomic annotation regions calculated by ChromHMM, each state was labelled with a functional annotation. Next, these 13 chromatin states were classified into five categories (enhancer, promoter, transcription repressed, heterochromatin, and quiescent) based on their function annotation. ChromHMM annotated all the 200‐bp intervals with a specific chromatin state across each stage in CRC development. With this, a *m* × *n* matrix represented all states were created, where *m* denoted the 200‐bp intervals and *n* is the time point. The state change across all time points was described for every 200‐bp interval within the entire genome using a Sankey diagram (https://plotly.com/python/sankey‐diagram/). A gene was assigned to a specific chromatin state when the 200‐bp bins annotated by this state had the highest frequency in ± 2 kb of the gene body region. This highest frequency state was defined as the dominate chromatin state of a gene.

### Dynamic Gene Analysis

To obtain dynamic expression genes across all time points in mouse CRC development, a time course regression analysis was conducted using maSigPro (version 1.58.0) implemented in R.^[^ ^37^ ^]^ In briefly, the expression of RNA was normalized to TMM by edgeR (version 3.32.1),^[^ ^38^ ^]^ and then fitted to a polynomial model with degree 4. The goodness‐of‐fit (R2) would be calculated for all genes, and only those with *R* ^2^ > 0.7 would be identified as dynamic expression genes. Next, a K‐means clustering analysis for these dynamic expression genes was performed using maSigPro, with the param k from three to nine. Four distinct clusters with obvious different expression patterns were gotten at last (Figure 3A).

In H3K27ac signal density dynamic analysis, the gene signal density was normalized to RPM within the region of the gene body ±1.5–10 kb. The dynamic H3K27ac signal density in genes was identified in the same way with RNA dynamic analysis. At last, nine distinct clusters were obtained.

### Transcript Factor Enrichment

For H3K27ac in each mouse CRC development stage, peaks calling by MACS2 across all replicates were merges to obtain a non‐redundant enhancer region list. Furthermore, the TSS ± 2 kb were removed to exclude an affect from the nucleosome free regions. Then motif enrichment analysis was performed with the regions in all five stages. The findMotifsGenome.pl module in HOMER (version 4.11, http://homer.ucsd.edu/homer/) was used to identify transcript factor motifs, with the size parameter 600 bp.

To investigate regulation network of the transcript factor Otx2, a public ChIP‐seq data of Otx2 (GSE56098) generated from mouse embryonic stem cells by Buecker et al. was selected.^[^ ^25^ ^]^ The bigwig file of Otx2 from their work was used to display the signal track in The UCSC browser view figures. To investigate regulation network of transcript factor identified, the transcript factor putative target gene list from Cistrome Data Browser was downloaded.^[^ ^39^ ^]^

### Correlation Analysis between Histone Modifications and RNA Expression

To evaluate the regulation of histone markers to gene RNA expression, the average signal density RPM for each marker across TSS ± 1.5 kb, TSS ± 1.5–10 kb and TSS ± 10–100 kb regions around the gene promoter was calculated. For OTX2 and NF‐*κ*B target genes, Pearson correlation coefficient in R between the gene expression level FPKM and marker's signal density RPM was calculated. In each time point, the gene's FPKM and RPM were averaged across all replicates.

### Gene Function Analysis

Differential expression genes detected in the 2‐, 4‐, 7‐, and 10‐week versus control were used to perform gene set enrichment analysis (GSEA) with R packages fgsea (version 1.16.0). Six CRC related pathways and five immune related pathways were selected to classify the sample stages. For the genes with different dominate chromatin state across CRC development stages, the chromatin state change profile between two stages was summarized. New enhancer state genes at 10‐weeks, original from quiescent or promoter state genes, were used in biological process analysis. Genes in each cluster identified by RNA dynamic analysis or H3K27ac dynamic analysis were used to conduct BP and KEGG pathway analysis, which was performed by R package clusterProfiler (version 3.18.1).^[^ ^40^ ^]^

### ChIP‐Seq and RNA‐Seq Data Visualization

UCSC genomic track for histone marks, RNA expression and chromatin state beyond the RefSeq gene model were drawn by karyoploteR,^[^ ^41^ ^]^ using the alignment file of chromatin markers and state annotation bed files produced by ChromHMM. Histone marker's signal density panel across TSS and gene body were plotted by in house R script, using the density matrix data produced by deeptools (version 3.3.2).^[^ ^42^ ^]^

### Reverse Transcription and Quantitative PCR

Cells were scraped down and collected with centrifugation. Total RNA was extracted with RNA extraction kit (Aidlab) according to the manufacturer's manual. Approximately 1 µg of total RNA was used for reverse transcription with a first strand cDNA synthesis kit (Toyobo). The resulted cDNA was then assayed with quantitative PCR. *β*‐actin was used for normalization. The sequences of primers are in Table S5, Supporting Information. Assays were repeated at least three times. Data were shown as average values ± SD of at least three representative experiments. *p*‐value was calculated using student's *t* test.

### Cell Proliferation Assay

The cell proliferation was measured using the MTT assay. Briefly, 1000 cells were seeded into 96‐well plate per well. Six well of each group were detected every day. MTT (0.25 µg) was put into each well and incubated at 37 ℃ for 4 h. The medium with the formazan sediment was dissolved in 50% DMF and 30% SDS (pH 4.7). The absorbance was measured at 570 nm. Assays were repeated at least three times. Data were shown as average values ± SD of at least three representative experiments and *p*‐value was calculated using student's *t* test.

### Transwell Assay

1 × 10^5^ HCT116 cells were plated in medium without serum or growth factors in the upper chamber with a Matrigel‐coated membrane (24‐well insert; pore size, 8 µm; BD Biosciences), and medium supplemented with 10% fetal bovine serum was used as a chemoattractant in the lower chamber. After 36 h of incubation, cells that did not invade through the membrane were removed by a cotton swab. Cells on the lower surface of the membrane were stained with crystal violet and counted. Assays were repeated at least three times. Data were shown as average values ± SD of at least three representative experiments and *p*‐value was calculated using student's *t* test.

### Xenograft Experiments in Mice

The 5‐week‐old male BALB/C nude mice were purchased from Beijing HFK Bioscience Co. Ltd. Colon cancer model was established by injecting subcutaneously 2 × 10^6^ HCT116 cells per site into the flank regions of the mice. Tumor volumes were measured twice a week using calipers. Tumor volumes were calculated as *V*  =  0.5 × length × width^2^. After 24 days of injection, the tumors were harvested and weighed.

### Availability of NGS Data

All the deep sequencing data generated in the current study have been submitted to GEO database, with the Acc. NO. GSE178145 (RNA‐seq) and GSE178144 (ChIP‐seq). Publicly available AOM/DSS RNA‐seq data are available under GEO Acc. NO. GSE57533. ChIP‐seq data for CRC patients are accessible at GEO Acc. NO. GSE156614. The remaining data are available within the article, Supporting Information, or available from the authors upon request.

### Code Availability

The code for all analyses presented in this paper is available from the authors upon request.

## Conflict of Interest

The authors declare no conflict of interest.

## Author Contributions

L.C., Z.L., and C.Z. contributed equally to this work. L.C. performed RNA‐seq, ChIP‐seq and most of the experiments; Z.L. and C.Z. performed bioinformatics analyses; Q.L. established CRC model and collected tissues; M.W., Z.‐X.C., and L.L. directed the project; M.W. and L.L. provided funding; M.W., L.C., Z.L., and C.Z. wrote the manuscript.

## Supporting information

Supporting Information

Supporting Table 1

## Data Availability

The data that support the findings of this study are openly available in GEO at https://www.ncbi.nlm.nih.gov/geo/query/acc.cgi?acc=GSE178214, reference number 178214.
