# Supplementary material for: Dynamic Chromatin States Coupling with Key Transcription Factors in Colitis‐Associated Colorectal Cancer
Source: Adv Sci (Weinh). 2022 Jun 16;9(23):2200536. doi: 10.1002/advs.202200536 (PMC9376751; doi:10.1002/advs.202200536)
Supplement: Supplementary file 1 — Supporting Information [file ADVS-9-2200536-s002.pdf]

ADVANCED SCIENCE
Open Access

Supporting Information

for *Adv. Sci.*, DOI 10.1002/advs.202200536

Dynamic Chromatin States Coupling with Key Transcription Factors in Colitis-Associated Colorectal Cancer

Lin Chen, Zhihui Luo, Chen Zhao, Qinglan Li, Yingjie Geng, Yong Xiao, Ming-Kai Chen, Lianyun Li, Zhen-Xia Chen and Min Wu**

Fig.S1

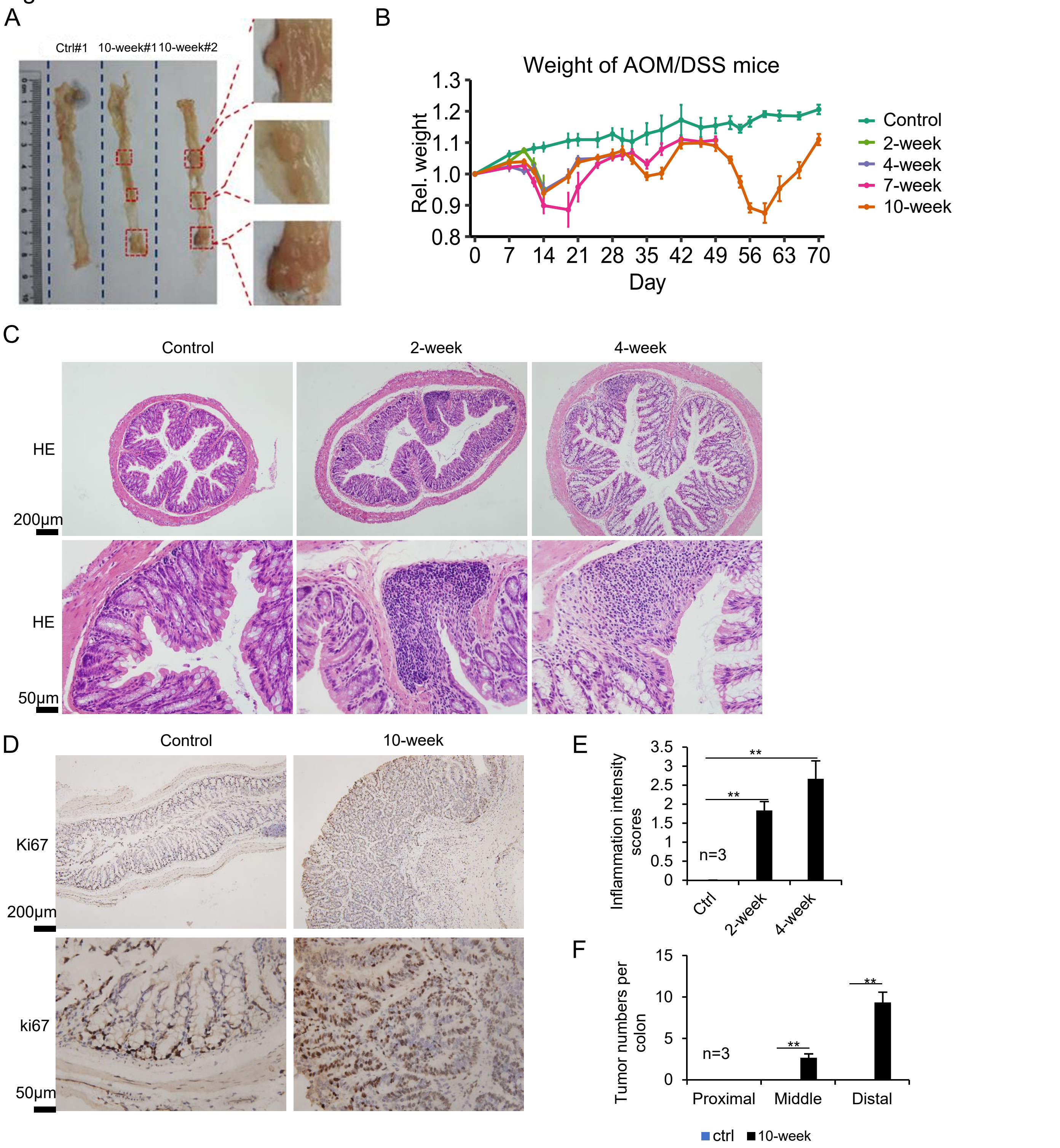

Sup Fig.S1 AOM-DSS mouse model was established. (A) Colorectum of control and 10-week group mice. There is no tumor in colorectum of the control mouse (control) and there are tumors in colorectum of the AOM-DSS treated mouse (10-week). **(B)** Mouse body weight measured during AOM-DSS mouse model building. Control group were fed with water as control. When fed with DSS solution, mice lost their body weight. When fed with distilled water, the body weight of mice recovered. **(C)** H&E staining of control, 2-week and 4-week mouse colorectums. **(D)** Ki67 staining of control and 10-week mouse colorectums. **(E)** The inflammation intensity scores of mouse colorectum (n=3). The scores were calculated by inflammation foci, crypt density and apoptosis crypt ratio showed in the H&E staining. **(F)** The colorectum were divided equally into three parts. The distal part was nearby anus and the proximal part was far away from anus. The figure shows tumor numbers of the three parts of colorectum (n=3). Data are represented as means ± SEM. Statistical significance was determined by unpaired 2-tailed Student's t test. ∗ p-value ≤ 0.05, ∗∗ p-value ≤ 0.01.

Sup. Fig. S2

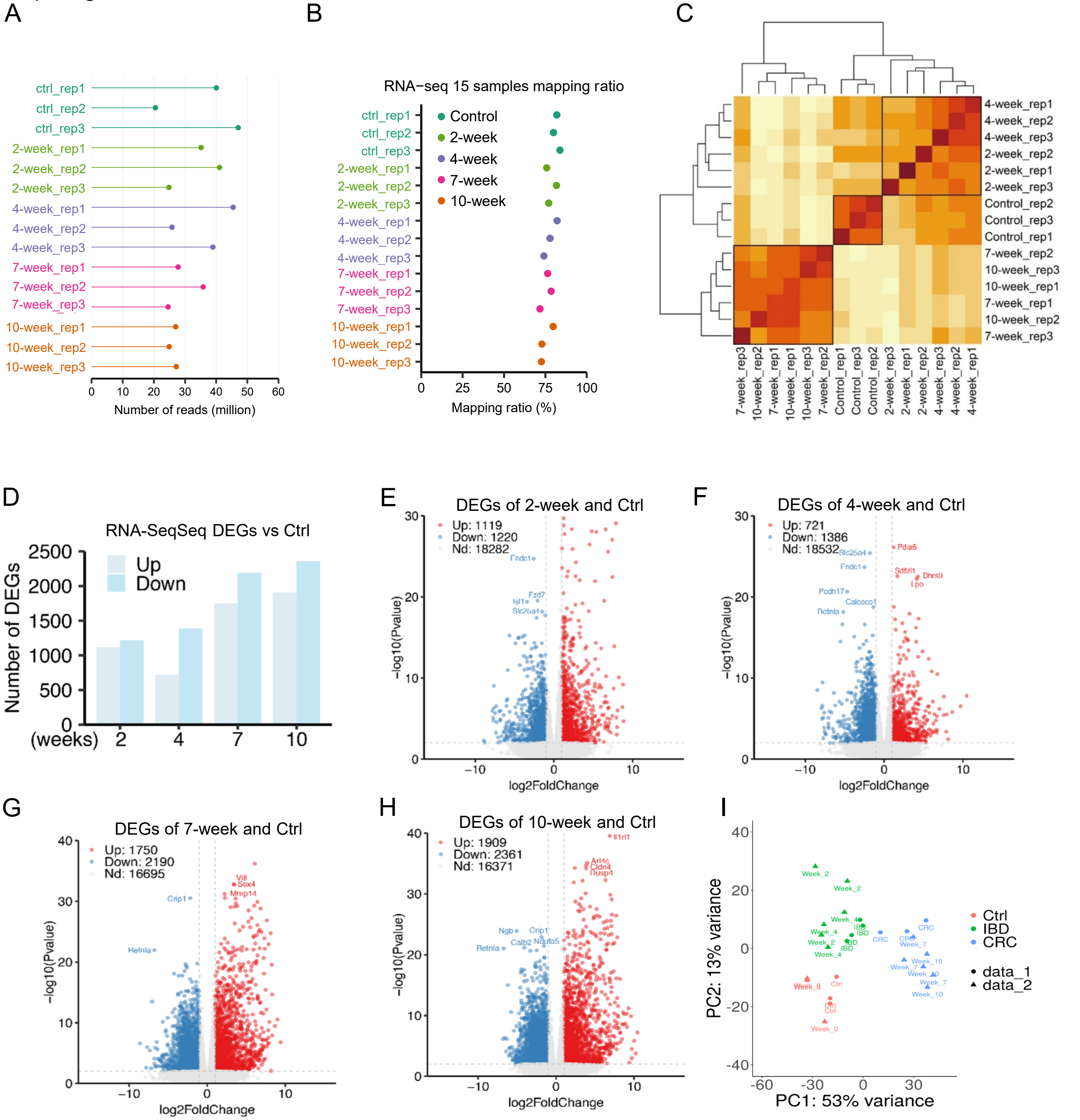

Sup. Fig. S2 Transcriptomic analysis of CRC tissues. (A) Read numbers of 15 RNA-seq samples. **(B)** Mapping ratios of RNA-seq 15 samples. Control, green; 2-week, light green; 4-week, purple; 7-week, pink; 10-week, orange. **(C)** Spearman correlation of RNA-seq data of 15 samples. The deeper color means a higher correlation. **(D)** DEG numbers of each time point vs control. **(E-H)** Volcano plots display DEG fold change (log2) between 2-week vs control (E), 4-week vs control (F), 7-week vs control (G), 10-week vs control (H). A threshold of 2 fold change and –log10 p-value is used for defining significant changes. Red dots represent up-regulated genes, blue dots for down-regulated genes and grey dots for genes not changed. **(I)** PCA using data of the current study and a previous report (Abu-Remaileh, Cancer research, 2015). Data1 represents previous report. data2 represents our current data.

Sup. Fig. S3

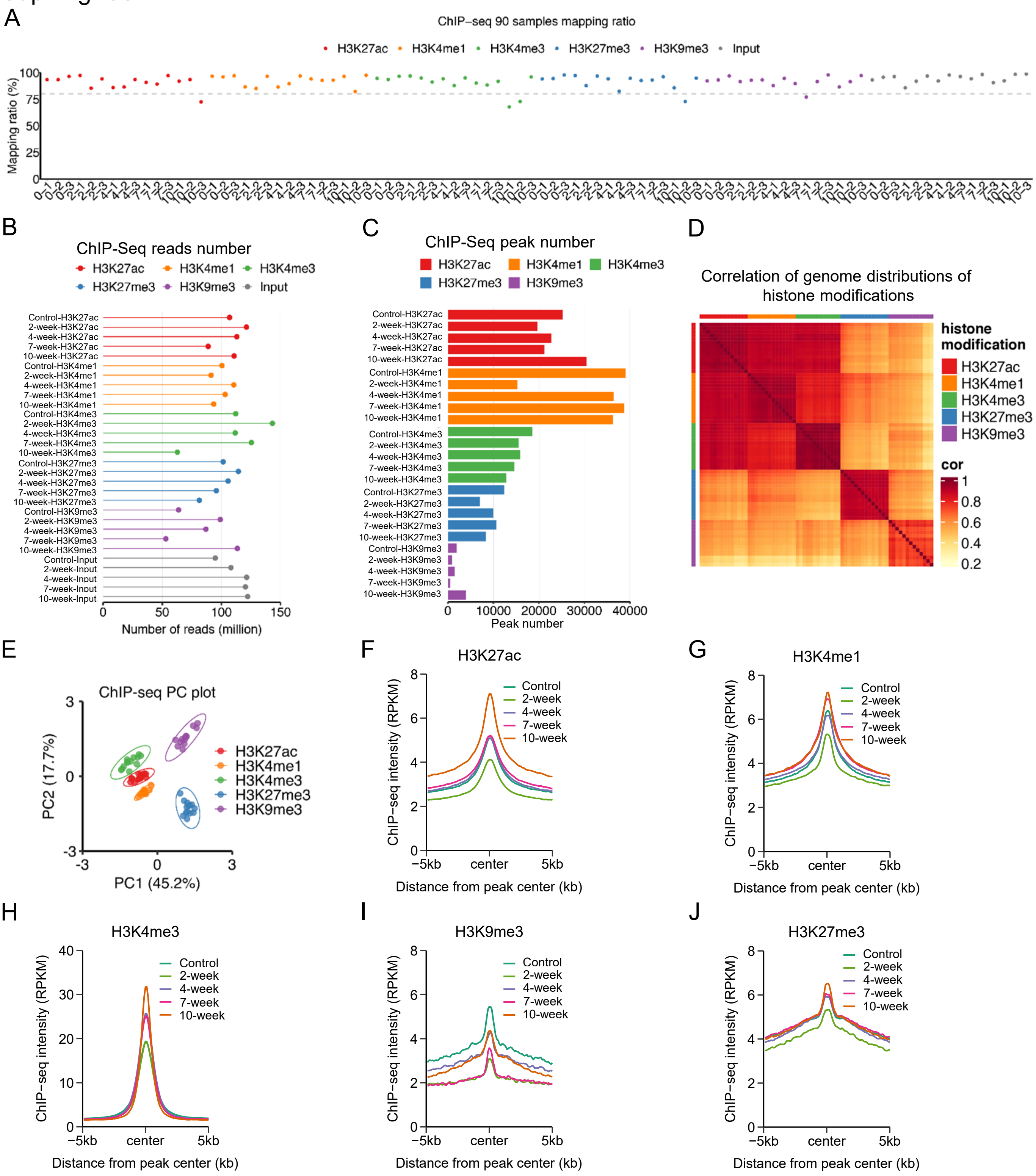

Sup. Fig. S3 Epigenomic analysis of CRC tissues. (A) Mapping ratios of ChIP-Seq samples (five kinds of histone modification and input at five-time points, total 90 samples). **(B)** Macs2 peak calling read numbers of ChIP-Seq samples. **(C)** Peak numbers of ChIP-Seq samples. **(D)** Spearman correlation of chip-seq data. The deeper color means a higher correlation. **(E)** PC analysis of ChIP-Seq data. **(F-J)** The ChIP-Seq intensity around TSS regions (TSS ± 5 kb) of H3K27ac (F), H3K4me1 (G), H3K4me3 (H), H3K9me3 (I), and H3K27me3 (J).

Sup. Fig S4

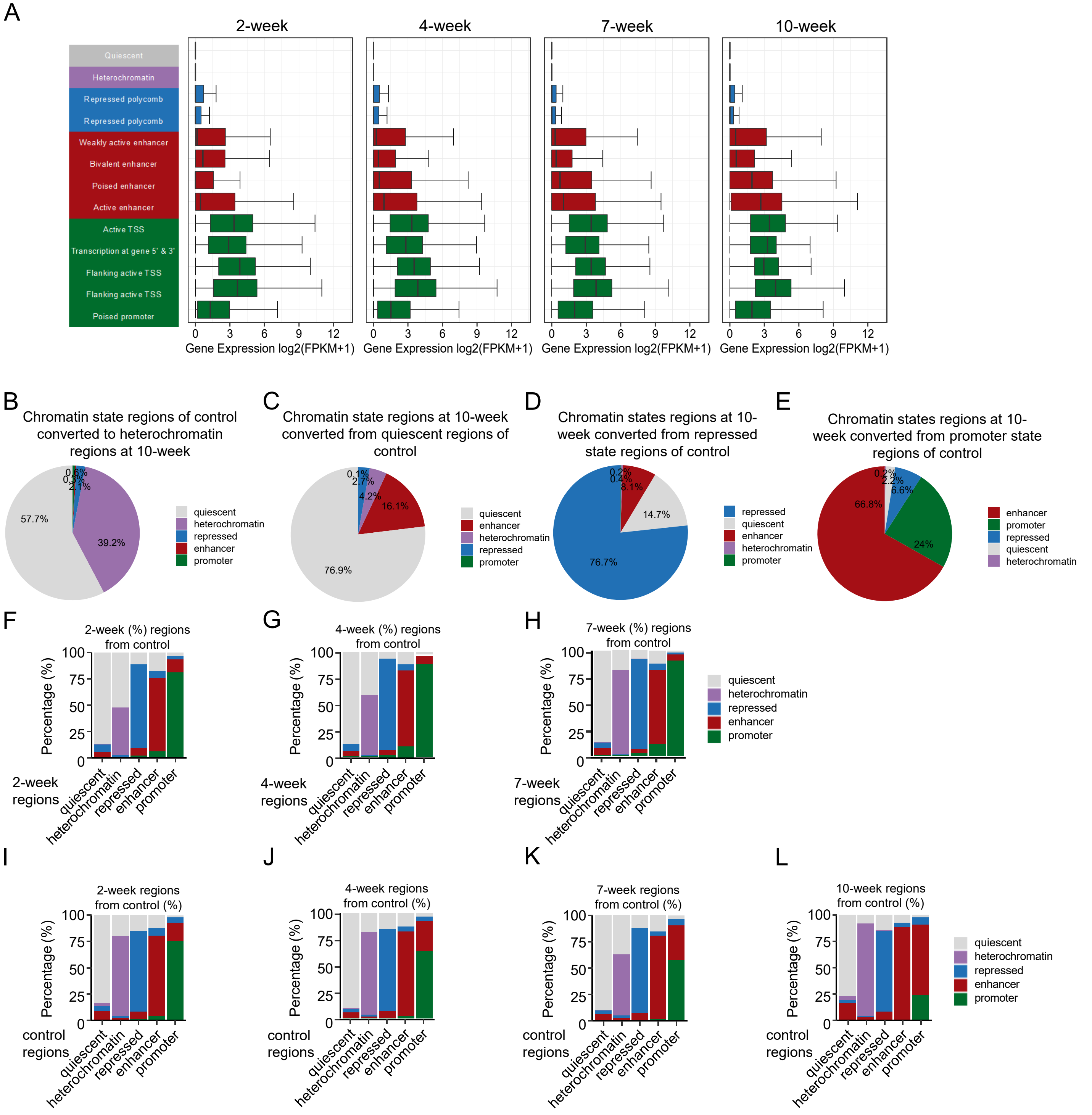

Sup. Fig. S4 Chromatin state dynamics during inflammation-cancer transition. (A) Average gene expression (FPKM) of genes within regions of particular chromatin states based on RNA-seq data at 2-week, 4-week, 7-week and 10-week. **(B)** Percentage of chromatin state regions at control converted to heterochromatin regions at 10-week. **(C-E)** Percentage of chromatin state regions at 10-week converted from control quiescent state (C), repress state (D) and promoter state regions (E). **(F-H)** Stacked bar charts display chromatin states regions (X axis) of 2-week (F), 4-week (G), and 7-week (H) converted from control regions (Colorful blocks). **(I-L)** The percentage of control chromatin state regions (X axis) converted to 2-week (I), 4-week (J), 7-week (K) and 10-week (L) (Colorful blocks).

Sup. Fig. S5

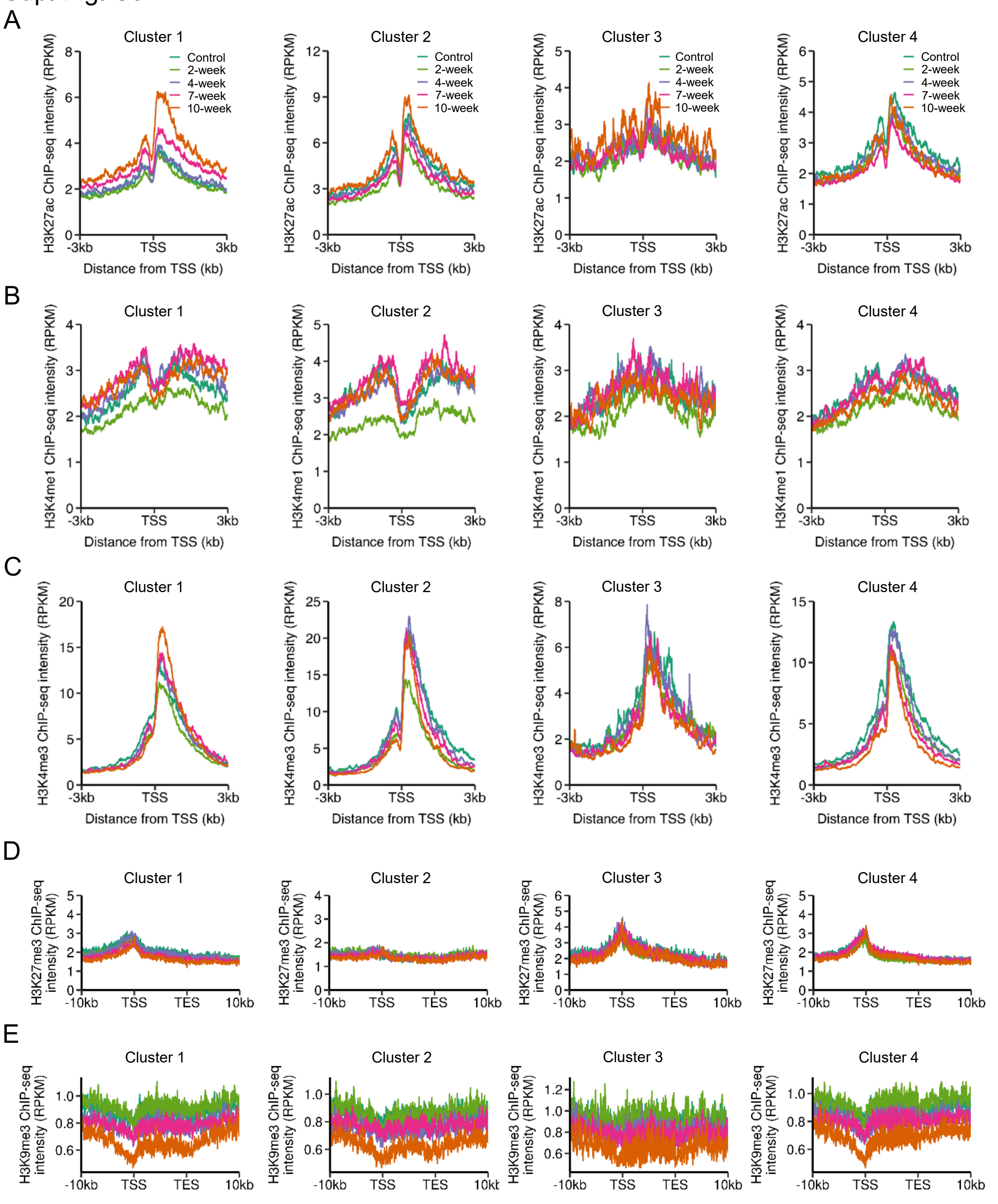

Sup. Fig. S5 ChIP-Seq intensity of histone modifications for DEGs in four functional gene clusters. (A-D) The average signals of H3K27ac (TSS ± 3kb, A), H3K4me1 (TSS ± 3kb, B), H3K4me3 (TSS ± 3kb, C), H3K27me3 (gene body ± 10kb, D) and H3K9me3 (gene body ± 10kb, E) enrichment of four RNA-seq clusters at five-time points. Green represents control, light green for 2-week, purple for 4-week, pink for 7-week, and orange for 10-week.

Sup. Fig. S6

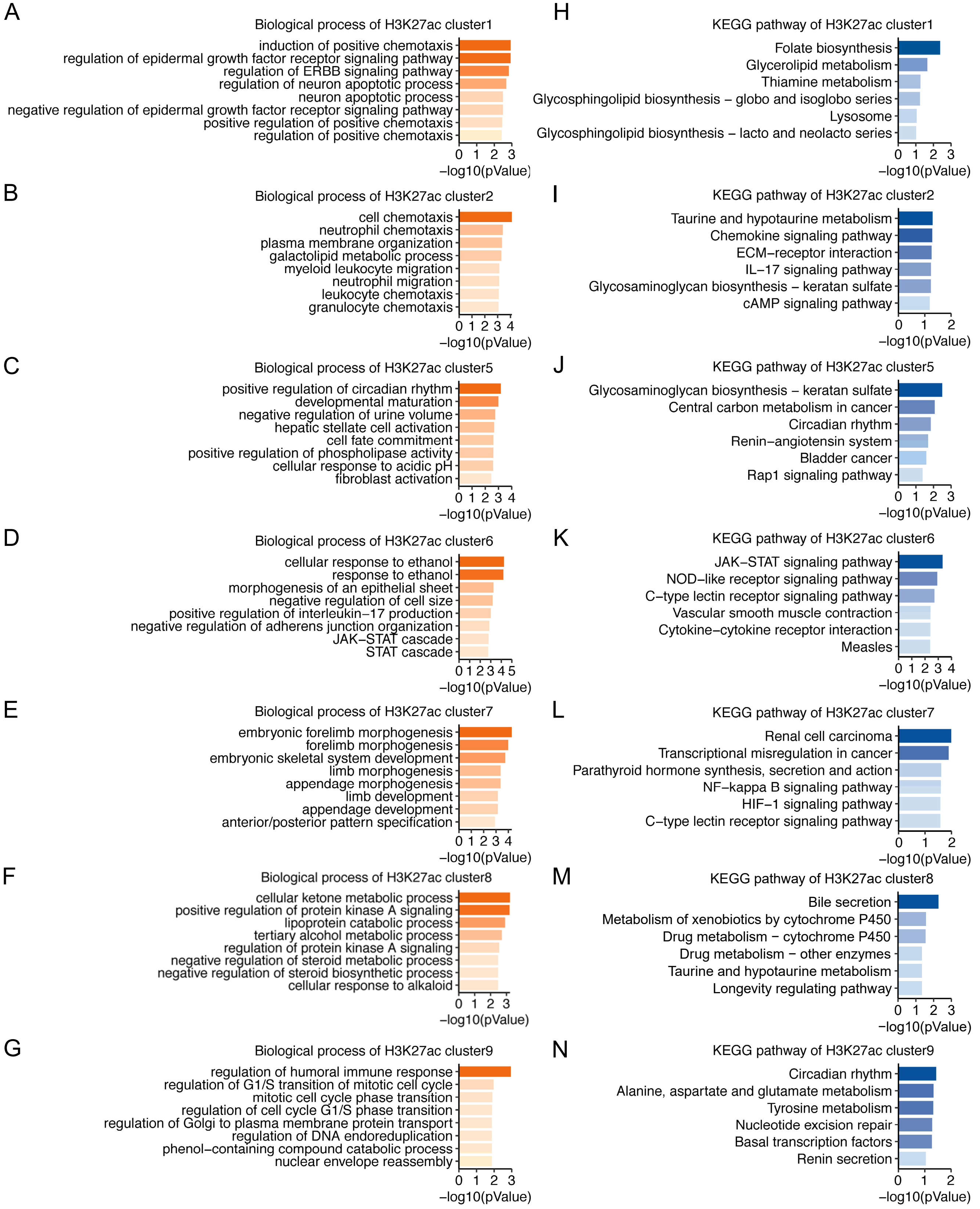

Sup. Fig. S6 Identification of DEG profile using maSigPro based on H3K27ac signal. (A-F) Biological process analysis of H3K27ac cluster 1 (A), 2 (B), 5 (C), 6 (D), 7 (E), 8 (F) and 9 (G) showed in figure 4A. **(G-L)** KEGG analysis of H3K27ac cluster 1 (H), 2 (I), 5 (J), 6 (K), 7 (L), 8 (M) and 9 (N) showed in figure 4A. Bar plot gradient color fill with p-value.

Sup. Fig. S7

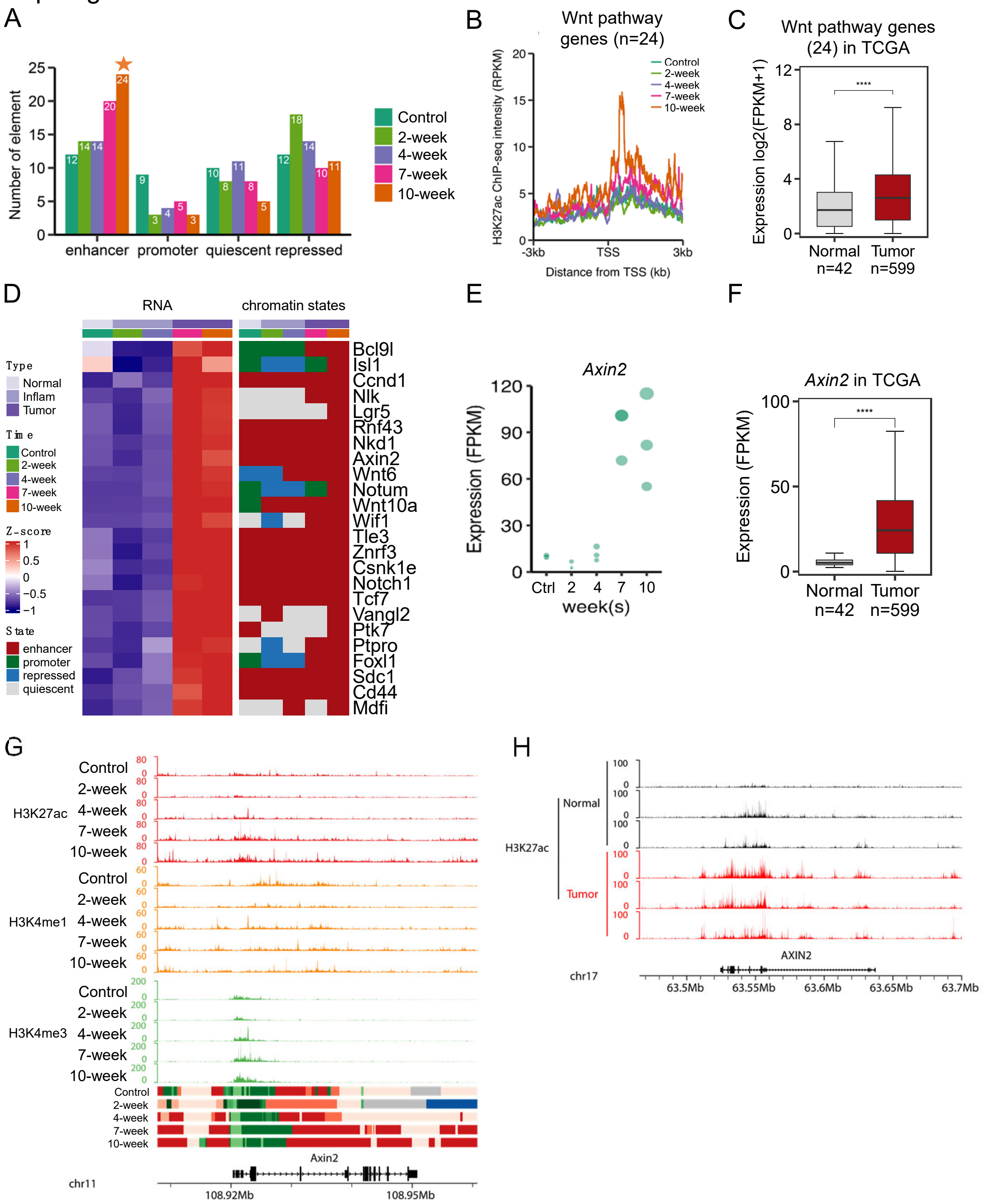

Sup. Figure S7 Enhancer state regions are associated with Wnt pathway gene expression. (A) The histogram shows chromatin states of Wnt signal pathway genes (n=44) belong to RNA-seq cluster 1 of Fig. 3A. **(B)** H3K27ac average signals (genebody ± 3 kb, RPKM) of Wnt pathway genes (n=24) which are with enhancer state at 10-week in (A). **(C)** Boxplots show average expression (FPKM) of Wnt signal genes (n=24) in tumor tissues (n=599) and normal tissues (n=42). The data sets of human colorectal cancer were downloaded from TCGA database. **(D)** Heatmap (left panel) displays the expression of Wnt signal genes (n=24) and their corresponding chromatin states at different time points (right panel). **(E&F)** *AXIN2* is shown as a representative gene. Its expression at five time points of AOM/DSS mouse model (E) and in patient tumor (n=599) and normal tissues (n=42) (F). **(G)** UCSC browser view for histone modifications and chromatin states of *AXIN2*. **(H)** UCSC browser view for H3K27ac enrichment on around *AXIN2* in 3 pairs of normal tissues and patient CRC tissues. Statistical analysis was performed using an unpaired Student's *t* test. $*$ $p < 0.05$, $**$ $p < 0.01$, $***$ $p < 0.001$, $****$ $p < 0.0001$, ns: no significance.

Sup. Fig.S8

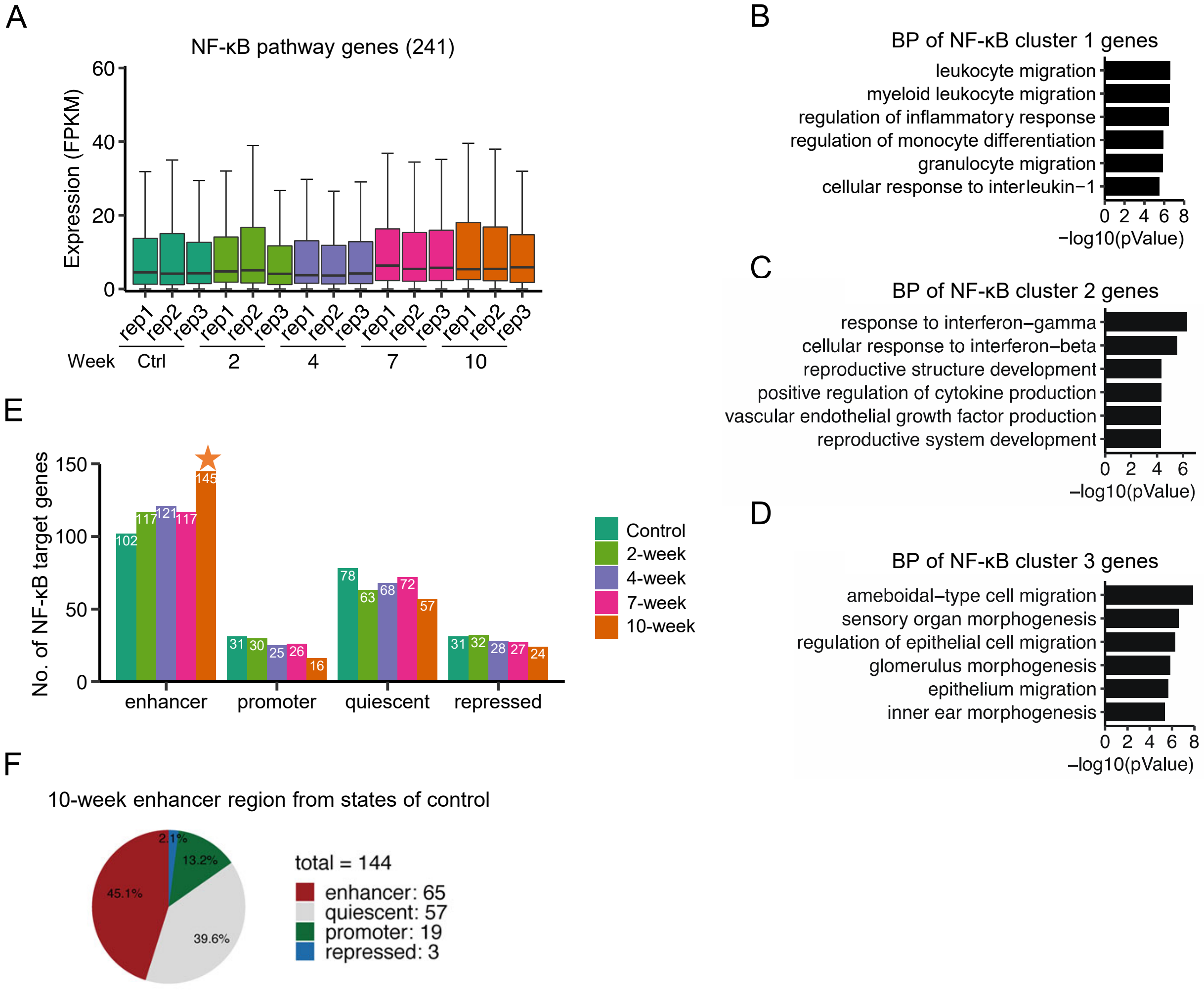

Sup. Fig.S8 Enhancer state is associated with selective activation of NF-κB pathway genes. (A) Expression of NF-κB pathway target genes expression in 15 samples of the mouse model. **(B-D)** Biological process analysis of genes in cluster 1 (B), cluster 2 (C) and cluster 3 (D) showed in figure 5A. **(E)** The histogram shows the chromatin states of NF-κB pathway genes (n=241) belong to H3K27ac cluster 4 (Fig. 4A) at five-time points. **(F)** The percentage of NF-κB downstream genes with enhancer state at 10-week (E) converted from control chromatin states.

Sup. Fig. S9

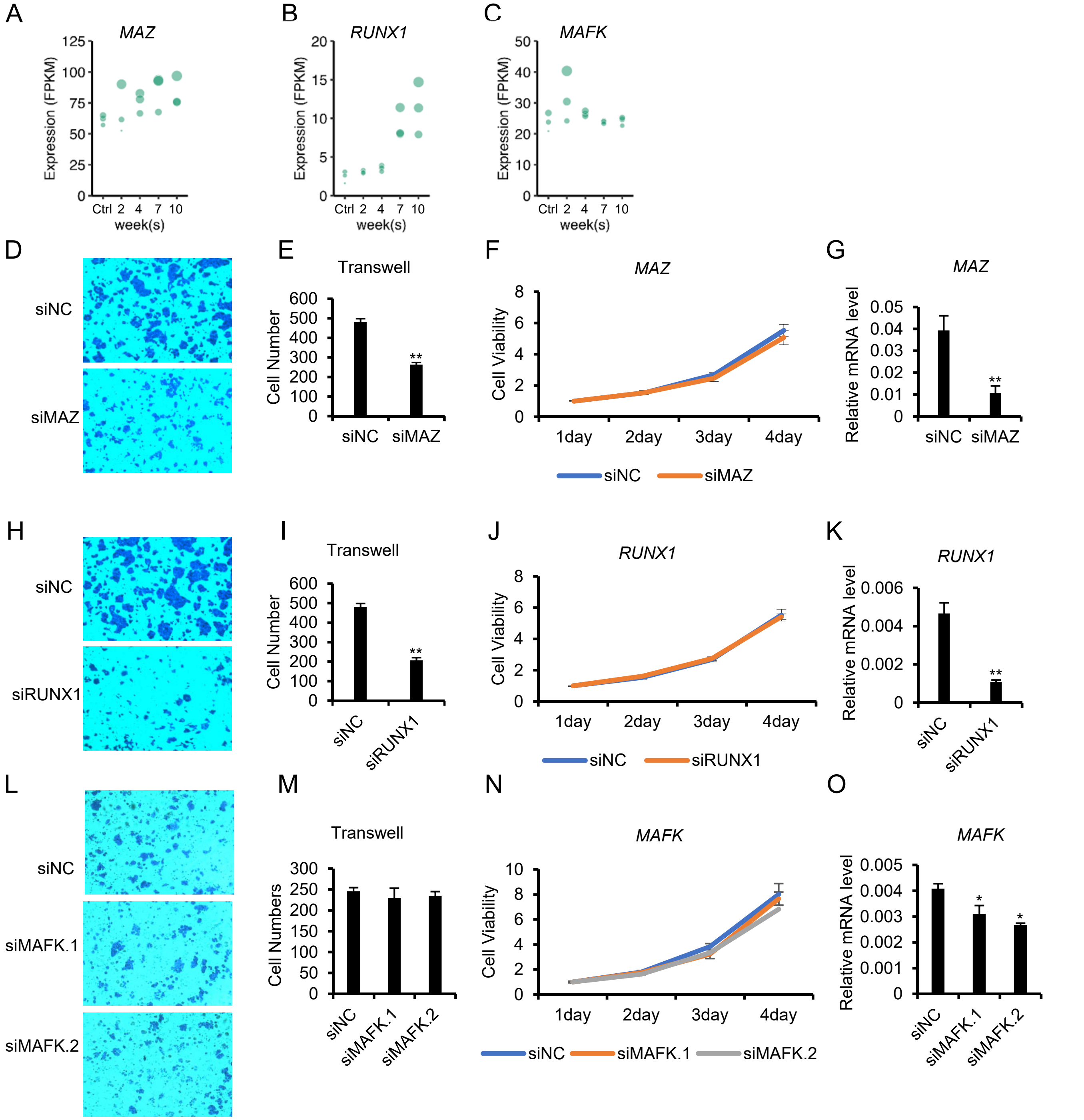

Sup. Fig. S9 Functional validation of predicted transcription factors in CRC. (A-C) *MAZ* (A), *RUNX1* (B) and *MAFK* (C) expression (FPKM) at five time points in AOM-DSS induced CRC tissues. **(D-O)** Transwell and cell survival analysis of HCT116 cells after knockdown of *MAZ* (D-G), *RUNX1* (H-K) and *MAFK* (L-O) by siRNAs. The results in all experiments represent the means (±SD) of at least three independent biological replicates. Statistical analysis was performed using an unpaired Student's t test. * means p-value ≤ 0.05, ** for p-value ≤ 0.01.

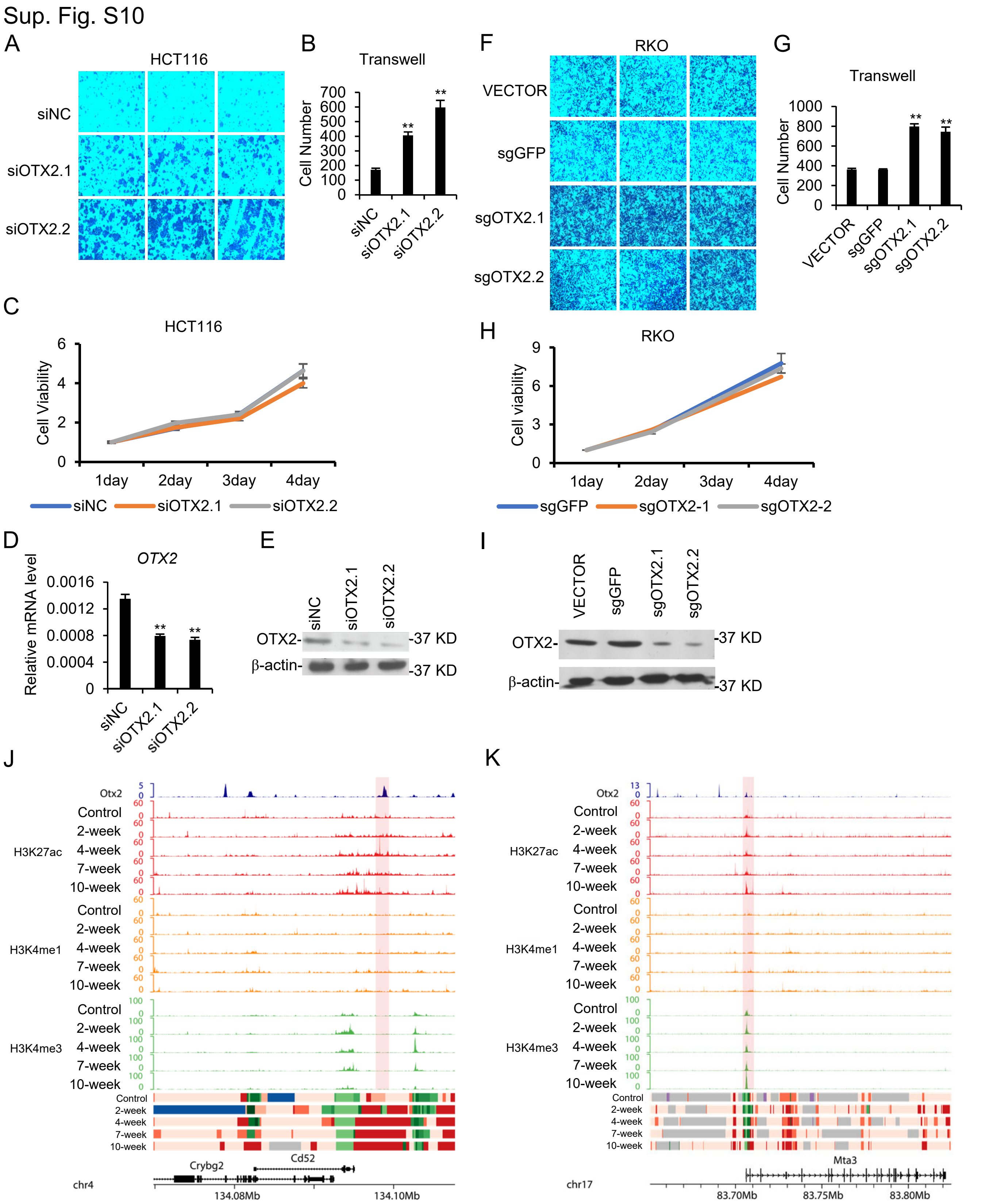

Sup. Fig. S10 Otx2 is a tumor suppressive transcription factor. (A-E) *OTX2* was knocked down by siRNAs in HCT116 cells. Cell migration was measured with transwell assay (A&B), and proliferation was measured with MTT assay (C). **(E-I)** *OTX2* was knocked down by sgRNAs in RKO cells. Cell migration was measured with transwell assay (F&G), and proliferation was measured with MTT assay (H). The results in all experiments represent the means (±SD) of at least three independent biological replicates. Statistical analysis was performed using an unpaired Student's *t* test. ∗ p-value ≤ 0.05, ∗∗ p-value ≤ 0.01. **(J&K)** The UCSC browser view to show Otx2 binding, H3K27ac, H3K4me1 and H3K4me3 enrichment around *Cd52* (J) and *Mta3* (K).
